# TGF-β inhibitor therapy decreases fibrosis and stimulates cardiac improvement in a pre-clinical study of chronic Chagas’ heart disease

**DOI:** 10.1371/journal.pntd.0007602

**Published:** 2019-07-31

**Authors:** Roberto Rodrigues Ferreira, Rayane da Silva Abreu, Glaucia Vilar-Pereira, Wim Degrave, Marcelo Meuser-Batista, Nilma Valéria Caldeira Ferreira, Otacílio da Cruz Moreira, Natália Lins da Silva Gomes, Elen Mello de Souza, Isalira P. Ramos, Sabine Bailly, Jean-Jacques Feige, Joseli Lannes-Vieira, Tania C. de Araújo-Jorge, Mariana Caldas Waghabi

**Affiliations:** 1 Laboratório de Genômica Funcional e Bioinformática—Instituto Oswaldo Cruz, Fundação Oswaldo Cruz (Fiocruz), Rio de Janeiro RJ, Brasil; 2 Laboratório de Biologia das Interações—Instituto Oswaldo Cruz, Fundação Oswaldo Cruz (Fiocruz), Rio de Janeiro RJ, Brasil; 3 Laboratório de Inovações em Terapias, Ensino e Bioprodutos—Instituto Oswaldo Cruz, Fundação Oswaldo Cruz (Fiocruz), Rio de Janeiro RJ, Brasil; 4 Departamento de Anatomia Patológica e Citopatologia, Instituto Nacional de Saúde da Mulher, da Criança e do Adolescente Fernandes Figueira, Fundação Oswaldo Cruz (Fiocruz), Rio de Janeiro, RJ, Brasil; 5 Laboratório de Biologia Molecular e Doenças Endêmicas, Instituto Oswaldo Cruz (FIOCRUZ/RJ), Rio de Janeiro, Brazil; 6 Laboratório de Virologia Molecular—Instituto Oswaldo Cruz, Fundação Oswaldo Cruz (Fiocruz), Rio de Janeiro RJ, Brasil; 7 UFRJ, Centro Nacional de Biologia Estrutural e Bioimagem, Rio de Janeiro, RJ, Brazil; 8 Université Grenoble-Alpes, Inserm, CEA, Biology of Cancer and Infection Laboratory, Grenoble, France; Instituto de Ciências Biológicas, Universidade Federal de Minas Gerais, BRAZIL

## Abstract

TGF-β involvement in Chagas disease cardiomyopathy has been clearly demonstrated. The TGF-β signaling pathway is activated in the cardiac tissue of chronic phase patients and is associated with an increase in extracellular matrix protein expression. The aim of this study was to investigate the effect of GW788388, a selective inhibitor of TβR1/ALK5, on cardiac function in an experimental model of chronic Chagas’ heart disease. To this end, C57BL/6 mice were infected with *Trypanosoma cruzi* (10^2^ parasites from the Colombian strain) and treated orally with 3mg/kg GW788388 starting at 120 days post-infection (dpi), when 100% of the infected mice show cardiac damage, and following three distinct treatment schedules: i) single dose; ii) one dose per week; or iii) three doses per week during 30 days. The treatment with GW788388 improved several cardiac parameters: reduced the prolonged PR and QTc intervals, increased heart rate, and reversed sinus arrhythmia, and atrial and atrioventricular conduction disorders. At 180 dpi, 30 days after treatment interruption, the GW3x-treated group remained in a better cardiac functional condition. Further, GW788388 treatment reversed the loss of connexin-43 enriched intercellular plaques and reduced fibrosis of the cardiac tissue. Inhibition of the TGF-β signaling pathway reduced TGF-β/pSmad2/3, increased MMP-9 and Sca-1, reduced TIMP-1/TIMP-2/TIMP-4, and partially restored GATA-6 and Tbox-5 transcription, supporting cardiac recovery. Moreover, GW788388 administration did not modify cardiac parasite load during the infection but reduced the migration of CD3^+^ cells to the heart tissue. Altogether, our data suggested that the single dose schedule was not as effective as the others and treatment three times per week during 30 days seems to be the most effective strategy. The therapeutic effects of GW788388 are promising and suggest a new possibility to treat cardiac fibrosis in the chronic phase of Chagas’ heart disease by TGF-β inhibitors.

## Author summary

TGF-β is a key molecule in many physiological processes as well as pathologies. We have previously described the role of TGF-β in Chagas disease, caused by the eukaryotic protozoan parasite *Trypanosoma cruzi*. Besides the high disease burden in many countries, one of the severe aspects of Chagas disease is the chronic heart condition developed by patients, for which there is no specific treatment. In search for a better treatment, we have investigated the potential of the TGF-β signaling blocker, GW788388, on disease hallmarks in a mouse model of chronic Chagas’ heart disease. Oral administration of GW788388 produces a global reversion of cardiac damage, and, remarkably, reduces fibrosis, one of the most important death-associated features in chronic Chagas’ heart disease.

## Introduction

Chronic chagasic cardiomyopathy (CCC) is the most common form of non- ischemic cardiomyopathy and one of the main causes of complications and death in Latin America, where the disease is endemic. It is estimated that ~7 million people are infected by *Trypanosoma cruzi* worldwide [1], but this is an underestimated screen as serology for Chagas disease is still not included in Public Health control programs in many countries. Chagas disease (CD) is caused by infection with *T*. *cruzi* parasites and presents an acute phase followed by a chronic phase, during which organ damage (mainly cardiac) can be observed in approximately one third of the patients [2].

CCC is a complex disease including host-parasite interactions contributing to an inflammatory and fibrotic scenario differing from other heart pathologies. As fibrosis is a major trait of CCC, specific anti-fibrotic therapies represent an alternative or complementary option to improve prognosis of this debilitating disease.

Transforming growth factor (TGF-β) is a pleiotropic cytokine with strong pro-fibrotic properties that has been shown to actively contribute to cardiac damage in several fibrotic disorders [3]. Interestingly, patients with atrial fibrillation present an overexpression of TGF-β in atrial tissue [4] and atrial fibrillation accompanied by myocardial fibrosis predisposes to arrhythmia events [5].

TGF-β is secreted under a latent form by almost all types of cells and needs to be activated into its mature form by different molecules such as thrombospondin, integrins or matrix metalloproteases [6]. To develop its biological functions, mature active TGF-β must bind to its membrane receptors, known as TGF-β receptor-type I (TβRI/ALK5) and -type II (TβRII). Ligand binding stimulates the phosphorylation of intracellular proteins of the classical pathway, Smad2/3, and some alternative pathways Erk, JNK, p38, PI3K [7].

We and others have previously demonstrated the involvement of TGF-β in CD physiopathology [8–12]. Chagas disease patients presenting more severe forms of heart disease progression have higher levels of circulating TGF-β [8, 11]. Single nucleotide polymorphisms in the TGF-β gene (-509 C<T and +10 T<C) were shown to be a risk factor for CD susceptibility, at least in the Latin American population [13,14]. Moreover, chronic chagasic patients with higher TGF-β levels present a worse clinical outcome after 10 years of follow up [15].

To understand the role of TGF-β in the physiopathology of CD, our group developed both *in vitro* and *in vivo* experimental models of the acute phase of the disease with important reproducible clinical features [16–19]. It was demonstrated that TGF-β favors *T*. *cruzi* cell invasion and the parasite intracellular cycle [16]. Data obtained *in vitro* with cardiomyocytes infected by *T*. *cruzi* and treated with anti-TGF-β compounds were confirmed in vivo, with an experimental model of acute phase infection [17–19], in which we observed reduced parasitemia followed by reduced cardiac damage and extracellular matrix deposition [18,19].

One century after the initial description of CD, therapy has made little progress and is still based on two trypanossomicidal drugs: nifurtimox and benznidazole (Bz). Recently, the BENEFIT randomized trail evaluated the efficacy of Bz on the clinical outcome of patients with CCC. This study showed a reduction of parasite load in serum but a lack of clinical effect on cardiac condition through a 5 year-follow-up [20]. The cardiac form of CD is characterized by progressive congestive heart failure with an inflammatory response, involving many cell populations such as CD4+ and CD8+ T cells, which triggers cardiac remodeling and myocardial fibrosis [21,22], associated with sudden cardiac death [23]. Current options for the treatment of CCC are based on generic therapeutic strategies that do not differ from those in other cardiomyopathies: diuretics, beta-blockers, angiotensin-converting enzyme inhibitors, and spironolactone [24–28]. A pre-clinical assay using mouse models of CCC has been performed using anti-inflammatory agents, such as anti-TNFα compounds [29].

In the present study, we have used a model of chronic Chagas disease [30–32] induced over several months following the injection of a low inoculum of *T*. *cruzi* (10^2^ parasites/mouse), to investigate whether GW788388 [33], an oral compound that inhibits TGF-β receptor kinase activity, could reverse heart fibrosis and electrical conduction defects. This model reproduces much more closely the human pathological situation than the acute infection models used in previous studies [18,19].

## Methods

### Ethics statement

All mice procedures were carried out in strict accordance with the recommendations in the Guide for the Care and Use of Laboratory Animals of the Brazilian National Council of Animal Experimentation (http://www.cobea.org.br/) and the federal law 11.794 (8 October 2008). Protocols used in this study were approved by the Institutional Committee for Animal Ethics of Fiocruz (CEUA/Fiocruz, Licenses LW10/14 and LW42-11). All efforts were made to minimize animal suffering.

### Mice, parasites and infection

Four- to six weeks old female C57BL/6 (H-2^b^) mice were obtained from the animal facilities of the Oswaldo Cruz Foundation (CECAL/Fiocruz, Rio de Janeiro, Brazil). Animals were housed for at least one week before parasite infection at the Cardoso Fontes Animal Facility/IOC under environmental factors and sanitation according to “Guide for the Care and Use of Laboratory Animals”. The Colombian strain of *T*. *cruzi* parasites was maintained by serial passage in mice every 35 days post-infection (dpi) and parasitemia was employed as a parameter to establish acute and chronic phases using 5 μL of blood obtained from the tail vein [29]. For all experimental procedures, C57BL/6 mice were infected by intraperitoneal injection of 100 blood trypomastigotes of the Colombian strain of *T*. *cruzi* [30].

### Drugs and treatment

The compound GW783388 (GlaxoSmithkline, France) or vehicle dilution buffer (4% DMSO, 96% [0.5% Hydroxypropylmethylcellulose (HPMC), 5% Tween 20, 20% HCl 1M in NaH2PO4 0.1M]) was used in oral administration. Mice received GW788388 at 3 mg/kg from 120 dpi, when electrical abnormalities and fibronectin deposition in the heart tissue are detected [30, 31], to 150 dpi by gavage in three administration schemes (0.2 mL): single dose and one or three administrations per week for 30 days. The control group received vehicle buffer using the same scheme.

### Experimental groups

Mice were divided into the following groups respecting the limit of 5 animals per cage: untreated non-infected (NI), Non-infected and GW788388-treated (NI+GW1x), infected and GW788388 untreated (*T*. *cruzi*) and infected and GW788388 treated, using 3 treatment schemes: single dose (SD); once (GW1x) and thrice (GW3x) a week.

### Electrocardiography (ECG) analysis

ECG recording and analysis were performed in all groups of infected and non-infected animals. Mice were intraperitoneally tranquilized with diazepam (20 mg/Kg), fixed in the supine position and the transducers were carefully placed subcutaneously according to chosen preferential derivation (DII). Traces were recorded using a digital system (Power Lab 2/20) connected to a bio-amplifier at 2 mV for 1 s (PanLab Instruments, Spain). Filters were standardized between 0.1 and 100 Hz and traces were analyzed using the Scope software for Windows V3.6.10 (PanLab Instruments, Barcelona, Spain). ECG parameters were recorded for at least 2 min and evaluated in the chronic phase at 120, 150 and 180 dpi, using the following standard criteria: the heart rate, monitored by beats/minute (bpm), and the variation at P wave and PR, QRS and correct QT intervals (QTc), all measured in milliseconds (ms). The ECG parameters were analyzed as previously described [29].

### Echocardiography analysis

For analysis of cardiac function, ECHO recording and analysis were performed in all groups. Mice were anesthetized (inhalation route) with 1.5% isoflurane gas in 100% oxygen with flow 1L/minute, trichotomized in precordial region and examined with a Vevo 770 ultrasound apparatus (Visual Sonics, Canada) coupled to a 30 MHz transducer. Left ventricular ejection fractions (LVEF) were determined using Simpson’s method and left and right ventricular areas (LV and RV) were obtained in B-mode using a short axis view at the level of the papillary muscles.

### TGF-β measurement

The estimation of TGF-β serum concentrations in samples of non-infected and infected animals at 120 and 150 dpi was performed using a TGF-β1 specific commercial enzyme linked immunosorbent assay (ELISA) kit (Quantikine TGF- β1 ELISA, R&D Systems, USA) according to the manufacturer’s instructions.

### Protein expression analysis

Extraction of protein from frozen heart tissue was performed as previously described [12]. Proteins were analyzed by immunoblotting with specific primary antibodies against SMAD2/3 (Cell Signaling– 8685), pSMAD2/3 (Cell Signaling– 3101), Fibronectin (Sigma–F3648), Collagen type 1 (Novotec, France, kindly provided by Dr. Daniella Areas Mendes-da-Cruz, IOC/Fiocruz), Timp-1 (Sigma–SAB4502971), Timp-2 (Sigma-AB2965), Timp-4 (Sigma- T8312). To confirm equal protein loading, the same membranes were stripped and reprobed with an antibody against GAPDH (Ambion–AM300).

### Gel zymography

40 μg of protein were loaded and separated on 12% SDS-PAGE with 0.1% gelatin incorporated as substrate. After running, gels were soaked in a sequence of baths (15 minutes in 2.5% Triton X-100 followed by 15 minutes in 2.5% Triton X-100 / 50 mM Tris-Cl pH 7.5 and 10 minutes, twice, in 50 mM Tris-Cl pH 7.5), under constant shaking. Gels were incubated overnight at 37°C in a 50 mM Tris-Cl pH 7.5 / 10 mM CaCl_2_ solution and then stained with 0.5% Coomassie brilliant blue R-250 and scanned in a GS-800 scanner (BioRad). The molecular masses of MMP-9 were estimated in Quantity One software (BioRad) by comparison with standards of PageRuler Plus Prestained Protein Ladder (Thermo Scientific).

### RNA and DNA extraction and Parasite load

In this study, RNA and DNA were extracted from the same heart tissue sample, using TRIzol. RNA and DNA were used to gene expression analysis and Parasite Load quantification, respectively. Following the TRIzol protocol, after the chloroform addition and separation in aqueous and organic phases, the aqueous phase was collected to extract RNA and DNA was extracted from the organic phase, following manufacture’s protocol. The parasite load estimation by qPCR was performed by absolute quantification, based on a standard curve produced from DNA samples extracted from 20 mg of heart tissue of a non-infected mice, spiked with 10^6^ parasites. The standard curve was built from the serial dilution of DNA, ranging from 10^6^ to 1 parasite equivalents. For parasite quantification, the qPCR reactions were carried out with 5 μL DNA; 10 μL FastStart Universal Probe Master Mix [2X] (Roche); 750nM cruzi1 (5′ASTCGGCTGATCG TTTTCGA3′) and cruzi2 (5′AATTCCTCCAAGCAGCGGATA3′) primers and 50nM cruzi3 probe (5′FAM-CACACACTGGACACCAA-NFQ-MGB3′), specific for the satellite region of the nuclear DNA of *T*. *cruzi*. In parallel, cardiac tissue amount was estimated by the quantification of mouse GAPDH, using the Pre-developed TaqMan Assay Reagents Mouse GAPDH [20X] (Applied Biosystems– 4352339E), at the final concentration of 1X, following manufacture’s protocol. The reactions were performed in ABI Prism 7500 Fast device (Applied Biosystems). The PCR cycling conditions were: a first step at 95°C for 10 min, followed by 40 cycles at 95°C for 15 s and 58°C for 1 min. The parasite load was calculated by *T*. *cruzi* equivalents/mice heart tissue equivalents ratio and expressed as “Parasite load/cardiac GAPDH”.

### Gene expression analysis

Extraction of total RNA from frozen heart tissue and the reverse transcription was carried out as previously described [34,12]. RT-qPCR was performed using TaqMan gene expression assays for MMP2 (Mm00439498-m1), MMP9 (Mm00442991-m1), GATA-4 (Mm00484689-m1), GATA-6 (Mm00802636-m1), Tbox-5 (Mm00803518-m1), Nkx2-5 (Mm00657783), Desmin (Mm00802455-m1), Titin (Mm00658612-g1), Troponin T (Mm00449089-m1) and the endogenous housekeeping control genes glyceraldehyde 3-phosphate dehydrogenase GAPDH (Mm99999915-g1) and β actin (Mm00607939-s1), which were purchased from Life Technologies (USA). The reactions were performed and analyzed as previously described [34].

### Histological assessment of cardiac fibrosis

Fixed tissue was dehydrated and embedded in paraffin. Sections (3 μm) were stained by Masson’s trichrome as previously described [19], for fibronectin, pSMAD2/3, connexin-43, Sca-1^+^ and DAPI detection by immunofluorescence and for CD3^+^ cells by immunohistochemistry analysis. Sections were observed using a Nikon microscope coupled with image acquisition systems (Nikon) and the images were assessed for percentage area of collagen using CellProfiler image analysis software (http://www.cellprofiler.org).

### Cytometric analysis of spleen cells

Mice spleen specimens were obtained from the all groups. Spleen samples were processed for flow cytometric analysis within 1h from harvesting, using a simple and rapid procedure. The tissue was first disrupted with mechanic process into cell culture medium. The cell suspension was then washed and resuspended in phosphate-buffered saline (PBS). Cell viability was assessed using Trypan Blue. For flow cytometric analysis, aliquots of cells were stained with saturating amounts of conjugated antibodies: CD4 (Southern), CD8 (BioLegend), CD44 (BioLegend), CD62L (BioLegend), CD49d (eBioscience), CD11a (Southern), CD45R (BioLegend). Samples were run and analyzed on a FACSCalibur instrument (BD Biosciences).

### Statistical analysis

Differences between infected and non-infected groups were considered statistically significant when ^$^p< 0.05, ^$ $^p<0.01, and ^$ $ $^P< 0.001 and differences between infected mice GW788388 treated or not are indicated by *P< 0.05, **P< 0.01, and ***P< 0.001, as determined by GraphPad Prism 4.0 software (GraphPad Software Inc., San Diego, CA, USA). All the analyses were performed using the non-parametric Mann–Whitney test.

## Results

The principal aim of the present study was to evaluate whether the TGF-β receptor inhibitor GW788388, reverses chronic cardiac fibrosis and improves heart electrical conduction in a well-established experimental model of chronic cardiomyopathy induced by *T*. *cruzi* infection, which reproduces relevant clinical features of chagasic heart disease, such as ECG and ECHO alterations and enhanced extracellular matrix deposition [30, 31]. In addition, the mechanism of action of this compound was also investigated. Based on previous data obtained from our group [19] using mice acutely infected by *T*. *cruzi*, we chose to administer GW788388 orally at the dose of 3 mg/kg.

### Set-up of the chronic model of *T*. *cruzi* infection

The chronic model of *T*. *cruzi* infection was set up as previously described [30, 31]. This experimental model of CCC uses a different parasite strain (Colombian, DTU-Tc I) and a more resistant mouse strain (C57BL/6) than in our previous experiments on acute infection [19]. C57BL/6 mice were infected by intraperitoneal injection of 100 blood trypomastigotes of the Colombian strain of *T*. *cruzi*. Infected mice were monitored by measuring the presence of circulating parasites during the peak of parasitemia at 42 dpi (as previously shown in 30). ECG parameters were evaluated in the chronic phase at 120 and 150 dpi, using the following standard criteria: (i) the heart rate was monitored by beats/minute (bpm), and (ii) the variation at P wave and PR, and QTc intervals, all measured in milliseconds (ms). ECG analysis demonstrated that at 120 dpi, all mice presented a significant decrease in heart rate, as measured by beats per minute (bpm) (S1A Fig), associated with significant increase of P wave duration (S1B Fig), PR (S1C Fig) and QT (S1E Fig) and QTc (S1F Fig) intervals and no difference in QRS interval (S1D Fig), when compared with sex- and age-matched non-infected (NI) controls. Atrioventricular block type 1 (AVB1) and type 2 (AVB2) events occurred in 80% of the mice (S1G Fig) and arrhythmia was observed in 100% of the infected animals (S1H Fig). Therefore, at 120 dpi the group of *T*. *cruzi*-infected mice show pivotal electrical abnormalities, as previously shown [30].

### Comparison of the different schemes of treatment with GW788388

We tested the effects of the GW788388 compound using three different administration schemes starting at 120 dpi: i) a single dose (GW); ii) one dose per week (GW1x) or iii) three doses (GW3x) per week during 30 days (until 150 dpi). Heart parameters were measured at the end of the experiment at 150 dpi. All treatment schemes improved P wave duration and PR interval (Table 1) whereas all but the single dose treatment decreased the prolonged QTc intervals (Table 1). In contrast, only the weekly treatments (GW1x and GW3x) were able to improve the heart rate (Table 1).

**Table 1:** ECG and ECHO observations.

	Mean value/ group ± SD					
ECG parameters	NI	NI+ GW 3x	Tc 150	Tc+ GW SD	Tc+ GW 1x	Tc+ GW 3x
Heart rate (bpm)	535±43	538±48.6	405±64^a^	429±83.6	471±45.4^d^	453±53^e^
PR intervals (ms)	39±2.7	38±2.1	46±2.8^a^	43±5.1^e^	42±1.5^d^	43±3.5^e^
P wave duration (ms)	10±0.8	11±0.2	14±1.5^a^	12.5±1.9^e^	11.9±2.1^d^	12±1.5^d^
QTc intervals (ms)	74±4.6	74±2.4	102±13.3^a^	99±6.2	91±8^e^	94±10.8^f^
Frequency of AVB1	0	0	100%	33%	33%	40%
Frequency of AVB2	0	0	80%	67%	40%	58%
LVEF (%)	62±4	64±4.6	51±5^a^	nd	60±3^f^	57±8.8
LV Volume (μl)	49±8	49±11	27±6^b^	nd	35±5.5^f^	45±22.7
FAC (%)	58±9	62±6.4	41.4±8^c^	nd	55±14^f^	45±15.3
LVID (mm)	3±0.2	3±0.3	3±0.2^b^	nd	3±0.1^f^	3±0.6
LV mass corrected (mg)	73±12	95±18	61±19^b^	nd	83±5^f^	72±32.4

GW788388 reverses many electrocardiogram (ECG) and echocardiogram (ECHO) abnormalities in mice chronically infected with *T*. *cruzi* (Tc 150). Significant differences between the values for non-infected (NI) and infected groups of mice

^a^(P< 0.001)

^b^(P< 0.01) and

^c^(P< 0.05). Significant differences between the values for infected non treated and treated groups of mice

^d^(P < 0.001)

^e^(P < 0.01) and

^f^(P < 0.05). For ECG analysis n = 16–18 mice per group and for ECHO analysis n = 6 mice per group.

SD = single dose; bpm = beats per minute; ms = milliseconds; AVB = Atrioventricular block; LVEF = Left ventricular ejection fractions; LV = Left ventricle; FAC = Fractional area change; LVID = LV internal diameter.

**Fig 1:**
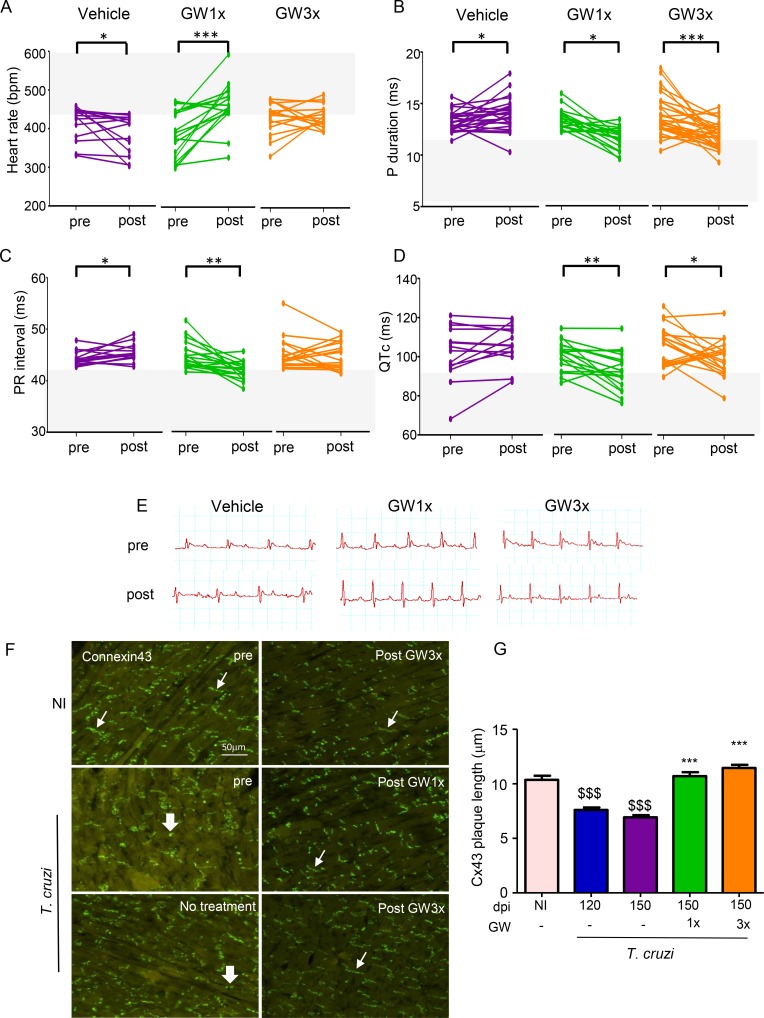
GW788388 reverses ECG abnormalities in a mouse model of chronic Chagas’ heart disease. Mice were infected with the Colombian *T*. *cruzi* strain (10^2^ parasites). Treatment with GW788388 started at 120 dpi until 150 dpi in two administration schemes: once a week (GW1x) and thrice a week (GW3x). Non-infected (NI) mice were monitored as a control group. Evolution of ECG intervals for each group of mice before (pre, 120 dpi) and after (post, 150 dpi) GW788388 treatment. Heart rate, in beats per minute (bpm) (A); P wave duration in milliseconds (B); PR interval in milliseconds (C); corrected QT interval in milliseconds (D); representative tracings for each group at 120 and 150 dpi (E). Asterisk indicates significant difference between mice before (120 dpi) and after treatment (150 dpi) (*P<0.05, **P<0.01, ***P<0.001). n = ~18 mice per group. GW788388 restores connexin 43 gap junction abnormalities in mice chronically infected with *T*. *cruzi* (F, G). At 120 and 150 dpi mice were sacrificed and heart sections were stained with anti-Cx43 antibody (green, Cx43 plaques are indicated by white arrows: thin small arrows indicate regular plaques and large arrows indicate disrupted plaques). Quantitative analysis of the length of Cx43 plaques (G) on images from each group studied. Significant differences between infected and non-infected groups are indicated by ^$ $ $^P<0.001 and differences between infected mice GW788388 treated or not are indicated by ***P<0.001 at 150 dpi. n = 4 mice per group.

In all further experiments, we only used two schemes for GW788388 treatment, once or thrice a week, for 30 days. When comparing the groups of mice before (at 120 dpi) and after the period of treatment (at 150 dpi), we observed that non-treated mice presented a significant decrease of heart rate and an increase in P wave duration, PR and QTc intervals (Fig 1A–1D). GW1x-treated mice presented a better cardiac rhythm, P wave duration, PR and QTc intervals (Fig 1A–1D) while GW3x-treated mice presented a significant decrease in P duration and QTc (Fig 1A–1D) as compare to non- treated mice. Importantly, we observed a reduction of AVB1 and AVB2 events after GW788388 treatment, as stated by ECG registers (Fig 1E). Six out of 18 mice (33%) treated with GW788388 once a week avoided sinus arrhythmia and 11 out of 18 mice (60%) reversed AVB2 events. From the group of mice treated with GW788388 thrice a week, 19 out of 30 mice (60%) avoided AVB1 events and 13 out of 30 mice (42%) avoided AVB2 events (Table 1).

Together, these data show that GW788388 treatment significantly improved heart function in infected mice. We next investigated the mechanism of action of this compound. We tested whether the positive effect on the heart rate could be mediated by rearrangement of gap junctions, more precisely connexin 43 (Cx43)-enriched plaques known for their importance in heart electrical conduction [35]. During the chronic phase of *T*. *cruzi* infection, mice treated with GW788388 once or three times per week presented better-organized Cx43-enriched plaque distribution (Fig 1F and 1G), possibly contributing to the improvement of electrical conduction (Table 1). Moreover, considering that Cx43 loss is established at 120 dpi [31], our data support that GW788388 therapy reverses this pattern.

### GW788388 administration did not modify the parasite load during *T*. *cruzi* chronic infection

**Fig 2:**
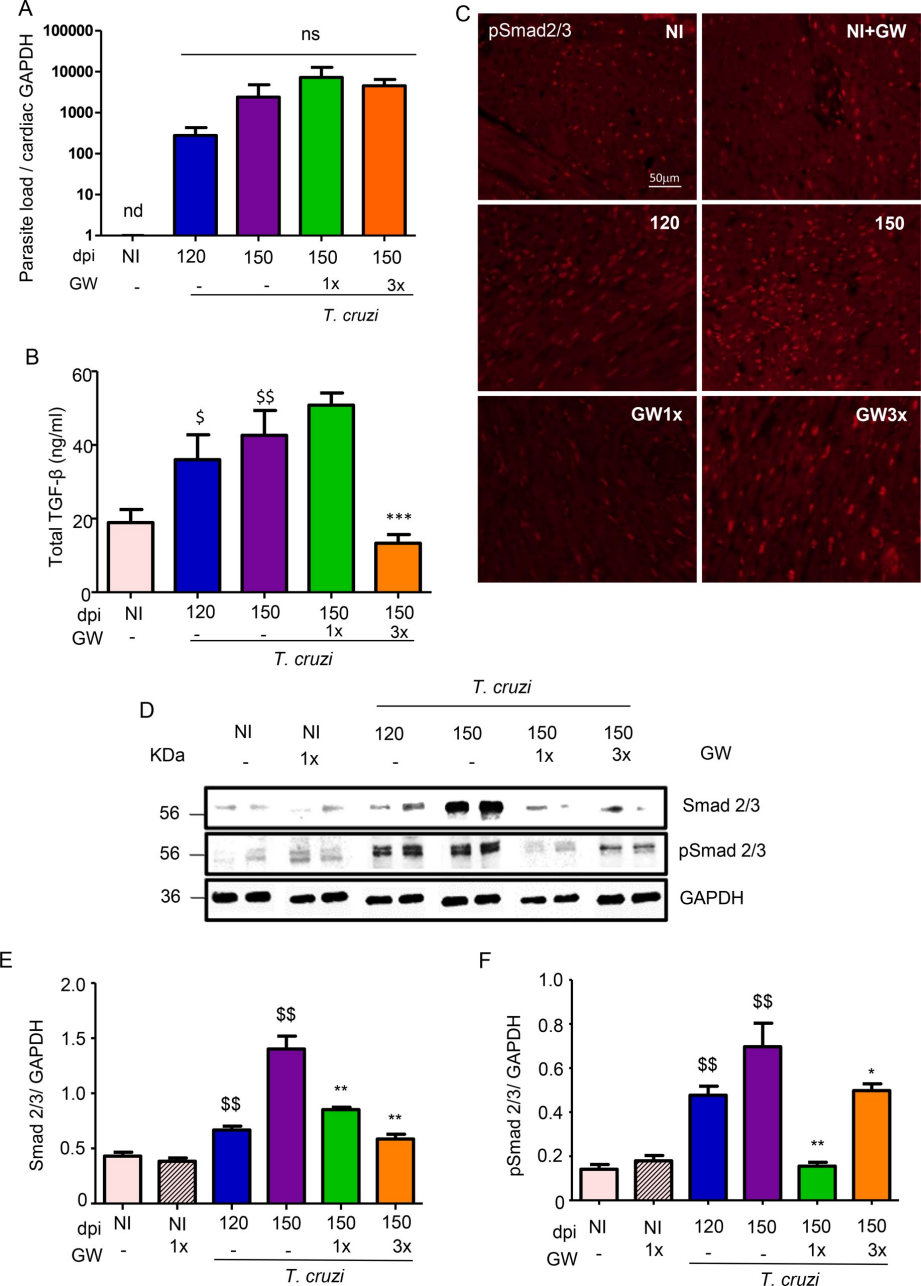
GW788388 decreases TGF-β expression and TGF-β activity but has no effect on parasite load in a mouse model of chronic Chagas’ heart disease. Mice were infected with *T*. *cruzi* from the Colombian strain (10^2^ parasites). Treatment with GW788388 started on 120 dpi until 150 dpi in two administration schemes: once a week (GW1x) and thrice a week (GW3x). Non-infected (NI) mice were monitored as a control group. (A) Parasite load is calculated by *T*. *cruzi* equivalents/mice heart equivalents ratio and expressed as “parasite load” (parasites eq./mg heart). Data represent mean± standard deviation of 6 mice per group in three independent experiments. (B) Total TGF-β levels measured by ELISA in the serum of mice. (C) Elevated pSmad2/3 staining (red, nuclear staining) in heart section of mice chronically infected with *T*. *cruzi*. Assessment of (D, E) expression and (D, F) phosphorylation of Smad2/3 in the heart of mice by Western blot analysis. Densitometric histograms of the normalized levels of Smad2/3 and pSmad2/3 normalized to GAPDH are shown. Data represent mean ± standard deviation of 6 independent mice per group in three independent experiments. Significant differences between infected and non-infected groups are indicated by ^$^P<0.05, ^$ $^P<0.01 and differences between infected mice GW788388 treated or not are indicated by *P<0.05, **P<0.01 and ***P<0.001. n = 6 mice per group.

As TGF-β has been described to influence *T*. *cruzi* cell invasion and the parasite intracellular cycle [17], in order to assess the effect of the inhibition of TGF-β pathway on parasite load, we verified *T*. *cruzi* DNA quantity from the heart tissue of infected mice. As expected, we observed the presence of *T*. *cruzi* DNA in the heart of chronic infected mice. Interestingly, both treatments with GW1x and GW3x at 150 dpi did not modify the parasite load, suggesting that the inhibition of TGF-β pathway does not interfere with *T*. *cruzi* control on chronic infection (Fig 2A).

### GW788388 administration decreases TGF-β expression and TGF-β activity

Recently, we have shown that *T*. *cruzi*-infected mice presented higher levels of circulating TGF-β during the acute phase of infection [12]. It is also known that TGF-β is involved in heart fibrosis of CD patients who present increased levels of serum TGF-β [8]. In the present mouse model of chronically Colombian-infected mice, levels of circulating TGF-β were increased at 120 dpi (p<0.05) and 150 dpi (2-fold increase; p<0.01). Although treatment with GW788388 once a week for four weeks did not impact TGF-β levels, GW788388 administration thrice a week from 120 dpi to 150 dpi significantly decreased TGF-β concentrations in serum (p<0.001) (Fig 2B).

Then, we verified whether the canonical TGF-β signaling pathway was activated in cardiac tissue after *T*. *cruzi* infection. We investigated the phosphorylation pattern of Smad2/3 in heart extracts. We observed that chronic infection by *T*. *cruzi* increased expression of total Smad2/3 and its phosphorylation, in the heart of infected mice as compared to non-infected animals (Fig 2C–2F). The treatment with GW788388 once or thrice a week decreased pSmad2/3 cardiac levels (Fig 2C, 2D and 2F) and the nuclear accumulation of pSmad2/3, indicating that the canonical TGF-β signaling pathway was down-regulated under these conditions (Fig 2C).

### GW788388 administration reverses heart fibrosis

**Fig 3:**
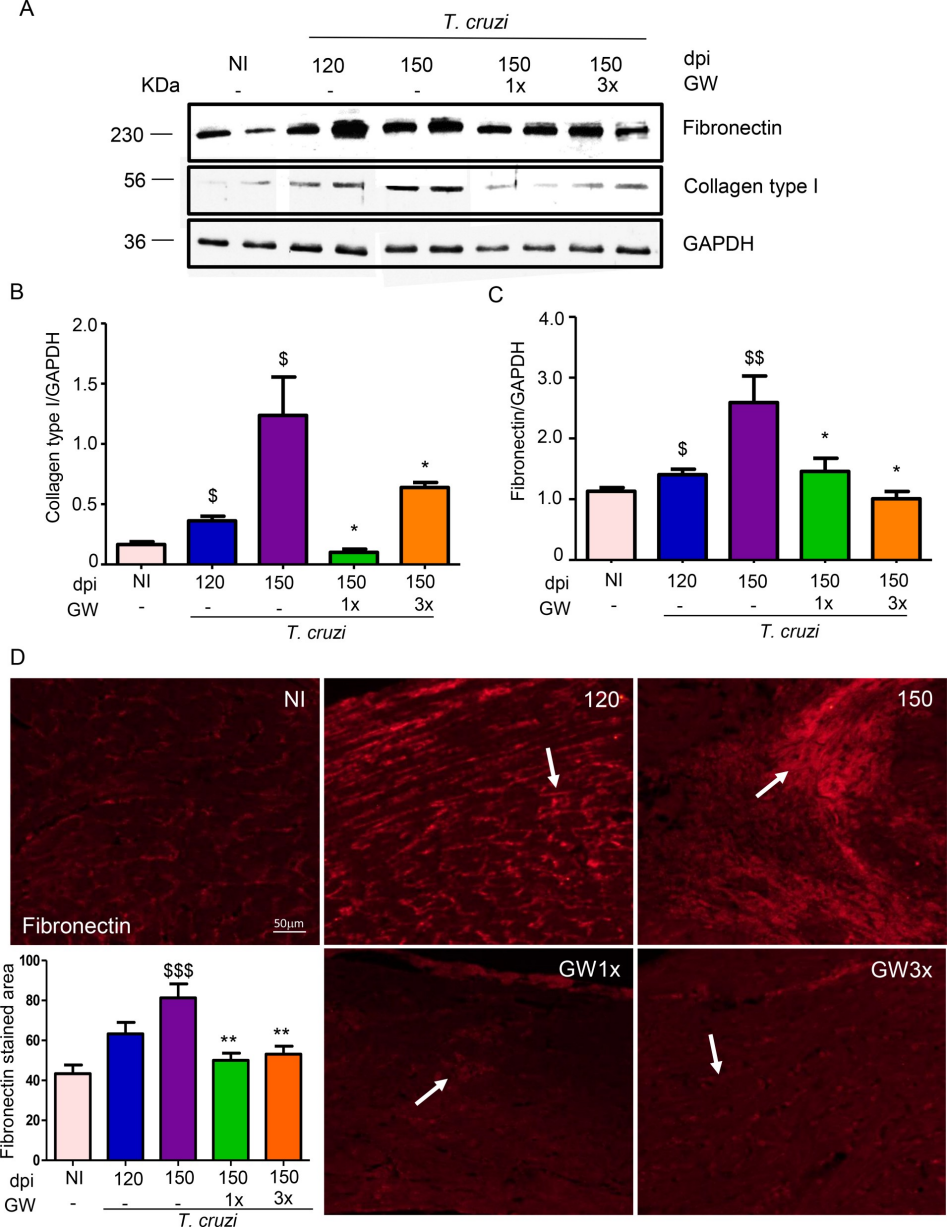
GW788388 decreases fibronectin and collagen type 1 expression in the heart of mice chronically infected with the Colombian *T*. *cruzi* strain (10^2^ parasites). Treatment with GW788388 started on 120 dpi until 150 dpi in two administration schemes: once a week (GW1x) and thrice a week (GW3x). (A) Western blot analysis of fibronectin and collagen type I protein expression in the heart. Densitometric histograms of the normalized levels of collagen type I (B) and fibronectin (C) normalized to GAPDH. n = 6 mice per group. (D) Heart sections were stained for fibronectin deposition (red staining, arrows). Fibronectin stained area were quantified on microscopic images of heart sections using Image J analysis software. Data is a representative image of 4–7 mice per group. Significant differences between infected and non-infected groups are indicated by ^$^P<0.05, ^$ $^P<0.01, ^$ $ $^P<0.001 and differences between infected mice GW788388 treated or not are indicated by *P<0.05 and **P<0.01.

**Fig 4:**
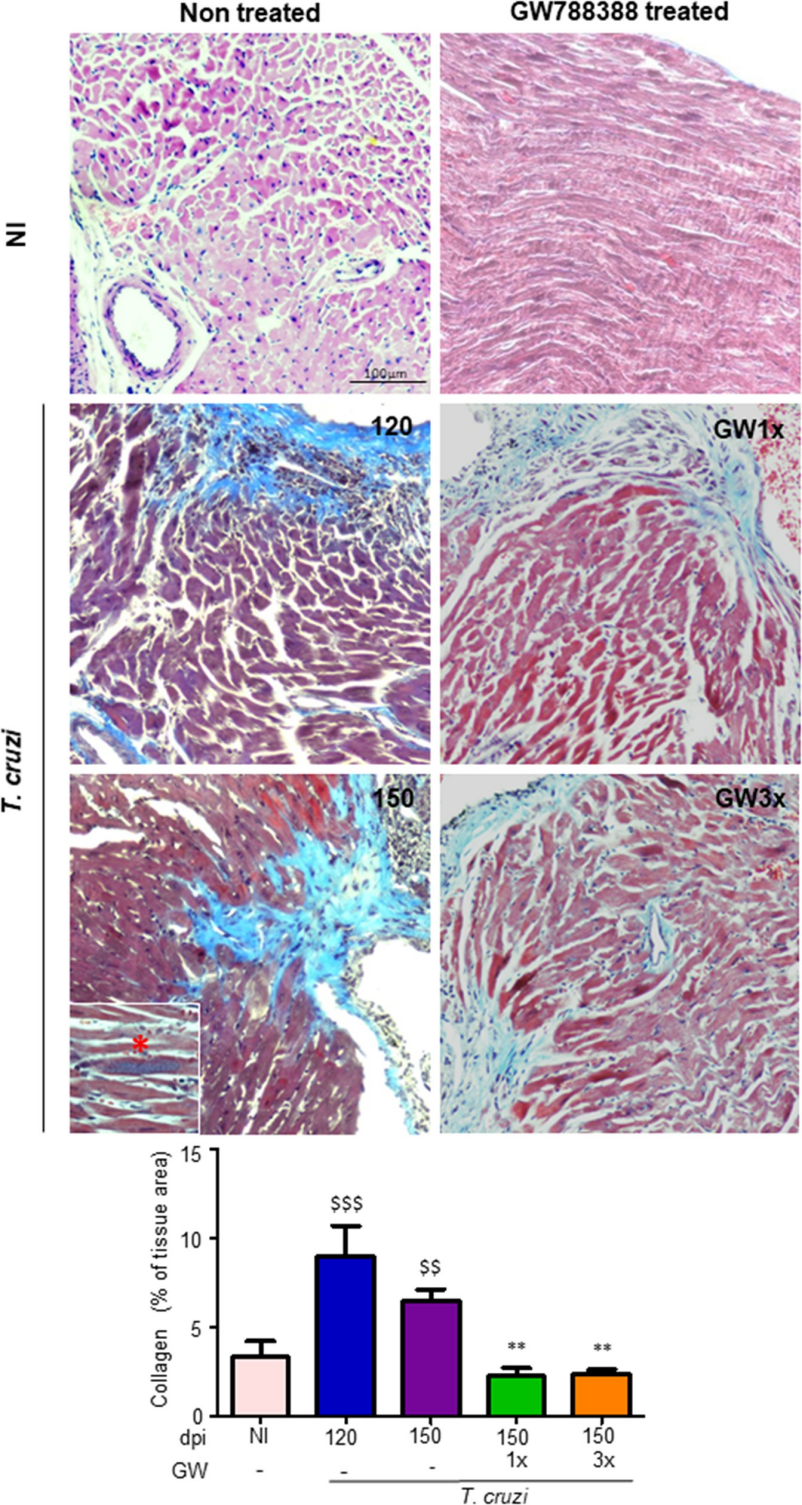
GW788388 treatment reverses heart fibrosis in mice chronically infected with *T*. *cruzi*. Mice were infected with the Colombian *T*. *cruzi* strain (10^2^ parasites). Treatment with GW788388 started on 120 dpi until 150 dpi in two administration schemes: once a week (GW1x) and three times a week (GW3x). Non-infected (NI) and infected mice were sacrificed at 120 and 150 dpi for fibrosis assessment. Heart sections were stained for collagen deposition by Masson’s trichrome (light blue staining). At 150 dpi it is possible to observe amastigotes nests (insert). Percent of Masson’s Trichrome stained area (light blue areas) were quantified on microscopic images of heart sections using CellProfiler image analysis software at 120 and 150 dpi. Data is a representative image of 10–15 independent mice per group. Significant differences between infected and non-infected groups are indicated by ^$ $^P<0.01, ^$ $ $^P<0.001 and differences between infected mice GW788388 treated or not are indicated by **P<0.01.

Our group previously demonstrated the involvement of TGF-β in the development of cardiac fibrosis due to CD, both in experimental models of *T*. *cruzi*-infected mice during the acute phase [12,18,19] and in patients during the chronic phase [8]. Here, we investigated the expression of the extracellular matrix proteins fibronectin and collagen type I and found an accumulation of both proteins in response to chronic *T*. *cruzi* infection in the ventricular heart tissue, observed at 120 and 150 dpi (Fig 3A–3D). Collagen expression was increased in the heart of infected mice as observed at 120 dpi and even more at 150 dpi. Interestingly, inhibition of TGF-β signaling by GW788388 administration using both schemes (once and thrice a week), significantly decreased extracellular proteins expression after 30 days of treatment, demonstrating its capacity to reverse cardiac fibrosis (Fig 3A–3D). Immunostaining for fibronectin (Fig 3D) and Masson´s trichrome staining for collagen deposition corroborated these data (Fig 4). We observed an increase in collagen deposition, visualized as light blue staining in the heart of the infected mice at 120 and 150 dpi as compared to non-infected animals (Fig 4). Moreover, GW788388 treatment clearly reduced collagen staining, suggesting cardiac tissue recovery. The process of fibrosis in response to a tissue damage could begin during the long-term injury stimulus, in which a loss of balance between the production and degradation of the ECM components is observed, leading to the gradual replacement of the functional tissue by a connective tissue [36]. On the other hand, decreased expression of extracellular matrix proteins could be associated to fibrosis reversion with replacement of functional tissue, indicating cardiac recovery.

### GW788388 administration improves cardiac function

**Fig 5:**
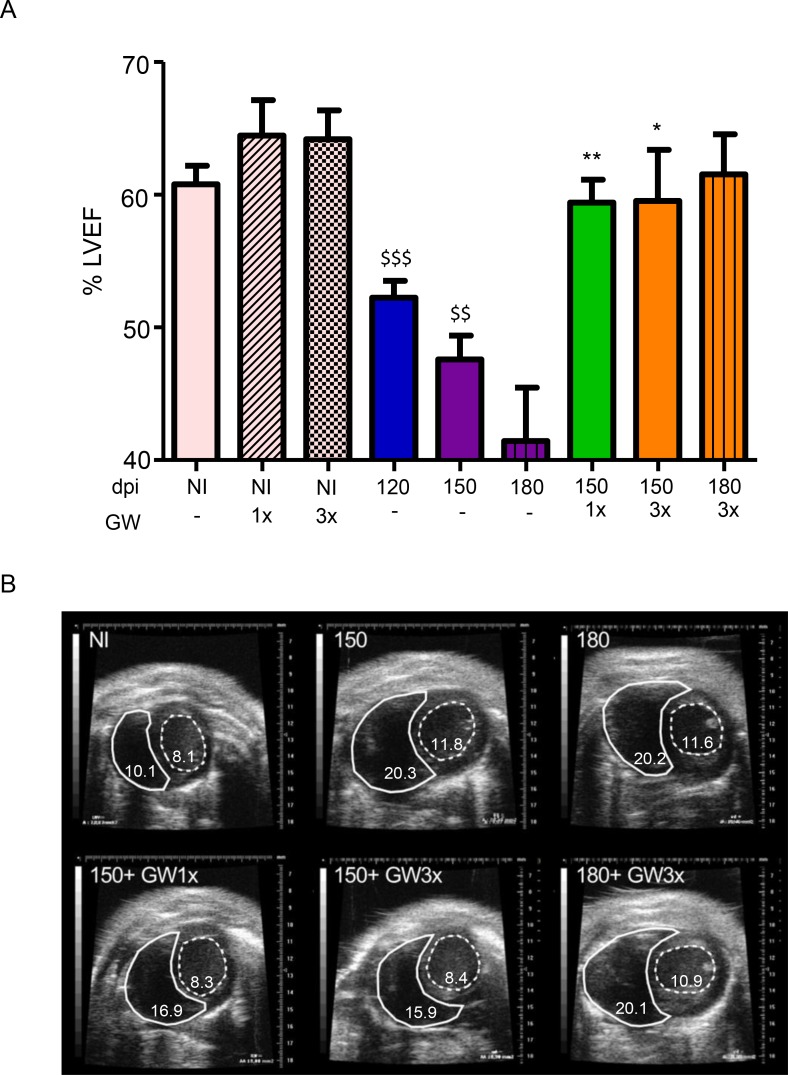
GW788388 treatment reverses heart-contraction in mice chronically infected with *T*. *cruzi*. Mice were infected with *T*. *cruzi* from the Colombian strain (10^2^ parasites). Treatment with GW788388 started on 120 dpi until 150 dpi administered once (GW1x) and thrice (GW3x) a week, followed up until 180 dpi. Echocardiography analysis showing (A) left ventricle ejection fraction (% LVEF); (B) right (continuous lines) and left ventricle (dashed lines) representative figure from each group of mice. Data represent mean± standard deviation of at least 4 mice per group. Significant differences between infected and non-infected groups are indicated by ^$ $^P<0.01 and ^$ $ $^P<0.001 and differences between infected mice GW788388 treated or not are indicated by *P<0.05 and **P<0.01. n = 2–6 mice per group.

Then, we aimed to observe if GW788388 treatment could also ameliorate cardiac function and structure by echocardiogram (ECHO). We investigated the percentage of the left ventricle ejection fraction (LVEF) by ECHO and observed that infected mice presented a reduced LVEF at 150 dpi (from 62,5% to 51,3%), corroborating previous data [32]. Importantly, GW788388 treatment once a week significantly reversed heart pumping to normal values (Fig 5A), reaching ~60% LVEF. We observed that other ECHO parameters were also altered, such as: i) LV internal diameter (LVID) and ii) LV stroke volume (Table 1). All parameters were significantly decreased at 150 dpi and GW treatment reversed to normal conditions (Fig 5A and 5B, Table 1). After GW788388 treatment cessation at 150 dpi, we followed up two different groups of *T*. *cruzi* infected mice non-treated and GW3x-treated for 30 days to verify whether therapy effects were sustained. At 180 dpi, we observed a low grade of LVEF in non-treated infected mice (~40%), while in the GW3x-treated group remained as the non-infected (~60%). Representative ECHO images from each group of mice are demonstrated in Fig 5B. n = 2–6 mice per group.

### GW788388 administration affect the expression/activity of two specific metalloproteinases

**Fig 6:**
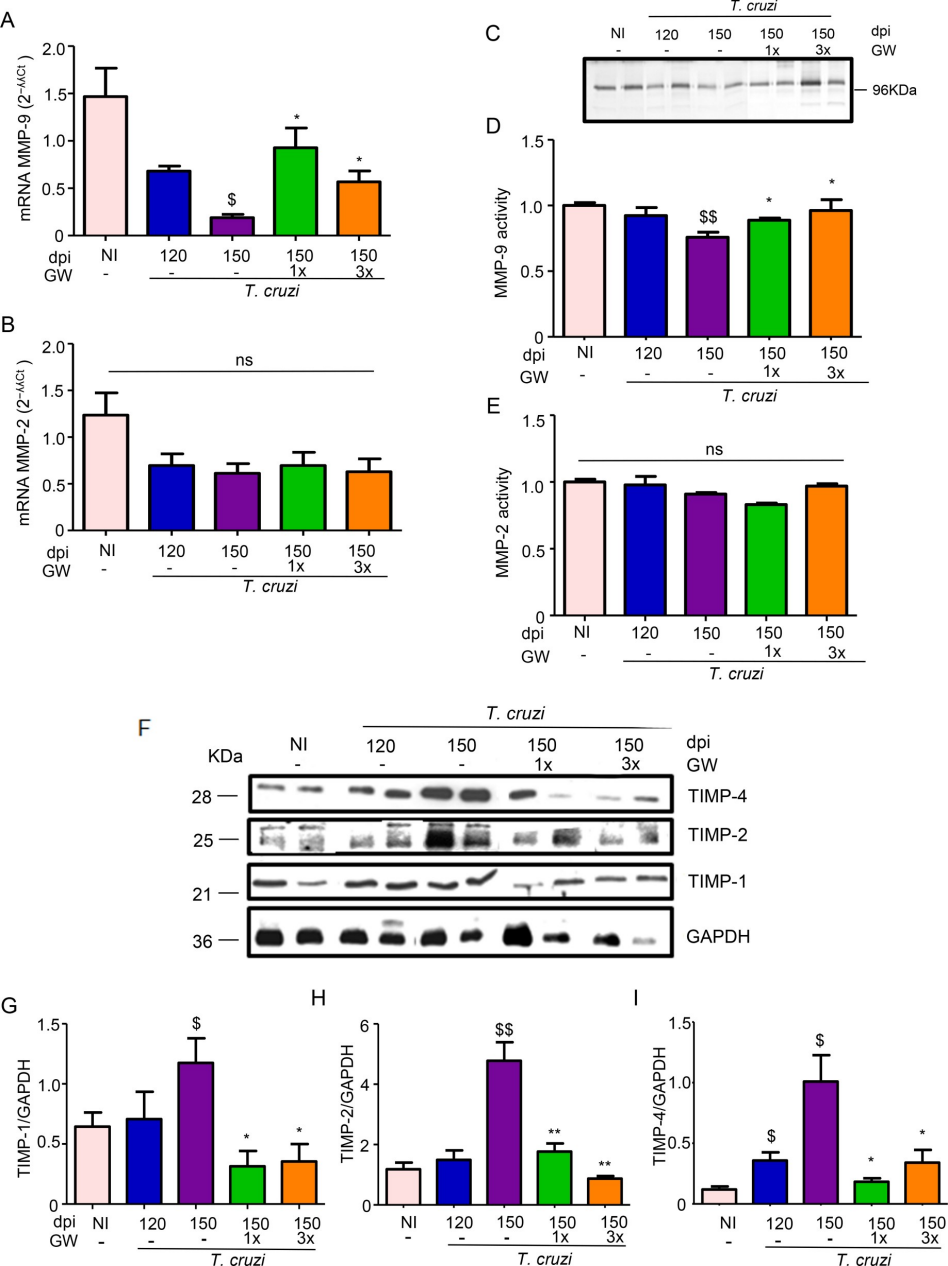
GW788388 treatment reverses MMP/TIMPs expression in the heart of mice chronically infected with *T*. *cruzi* after GW788388 treatment. Mice were infected with the Colombian *T*. *cruzi* strain (10^2^ parasites). Treatment with GW788388 started on 120 dpi until 150 dpi in two administration schemes: once a week (GW1x) and three times a week (GW3x). Non-infected (NI) mice were monitored as a control group. (A) MMP-9 mRNA levels, (B) MMP-2 mRNA levels relative to GAPDH levels from hearts of infected mice (2^−ʎʎCt^ values, fold change) are plotted. (C) Representative zymography gel showing the gelatinolytic activity of MMP-9; Densitometric histogram of the mean values of detected gelatinolytic activities from (D) MMP-9 and (E) MMP-2. (F) Assessment of TIMP-1, TIMP-2 and TIMP-4 expression in the heart by Western blot analysis. Densitometric histograms of the normalized levels of TIMP-1 (G) TIMP-2 (H) and TIMP-4 (I) in relation to GAPDH are shown. Data represent mean± standard deviation of 6 mice per group in three independent experiments. Significant differences between infected and non-infected groups are indicated by ^$^P<0.05 and ^$ $^P<0.01, and differences between infected mice GW788388 treated or not are indicated by *P<0.05 and **P<0.01. n = 4–6 mice per group.

To investigate the possible mechanisms involved in the reversal of heart electrical and functional abnormalities and fibrosis, we evaluated the involvement of the metaloproteinases MMP-2 and -9 activities in this process. At 150 dpi, *T*. *cruzi* infection significantly reduced MMP-9 mRNA expression and activity, (Fig 6A, 6C and 6D). GW788388 treatment, in the two schemes (once or thrice a week), significantly increased MMP-9 mRNA levels and its enzymatic activity (Fig 6A, 6C and 6D). On the other hand, MMP-2 transcription and activity were not affected (Fig 6B and 6E).

As metaloproteinases are highly regulated and their transcript levels cannot be directly correlated to their activity, we investigated some of the most important MMPs regulators such as TIMP-1, -2 and -4. In order to understand the differences in MMPs expression and activities, we verified if one of the intrinsic regulators of MMPs, tissue inhibitor of matrix metaloproteinases -1, -2 and -4 (TIMP-1, TIMP-2 and TIMP-4) presented altered protein expression in the heart, directly by Western blotting assays. We demonstrated that chronic *T*. *cruzi* infection induced the expression of TIMP-1, TIMP-2 and TIMP-4 in heart tissue (Fig 6D–6G). In GW788388-treated infected mice, both schemes significantly reduced the expression of TIMP-1 (~45%), TIMP-2 (~65%) and TIMP-4 (~80%) (Fig 6D–6G).

### GW788388 administration increases cardiac recovery

**Fig 7:**
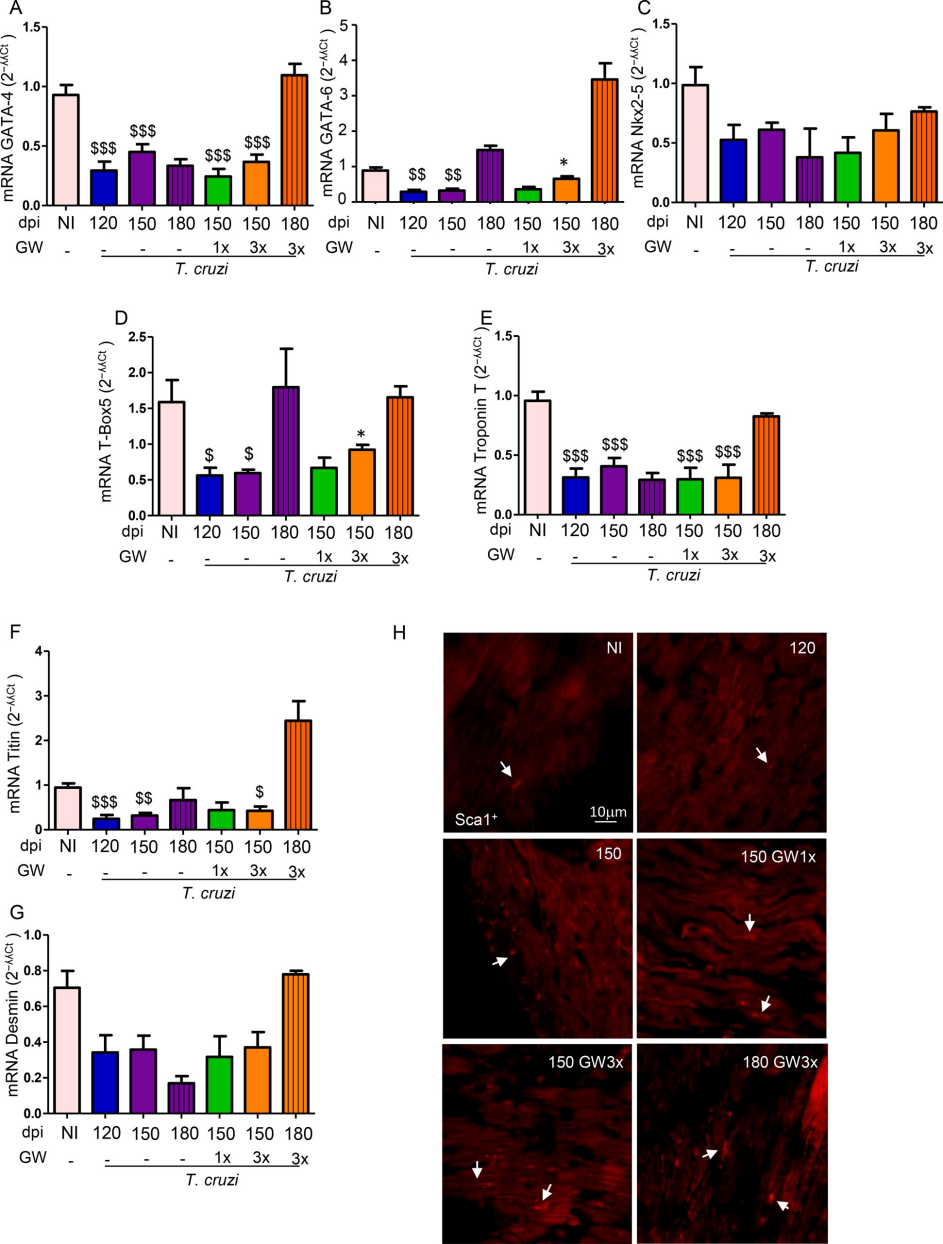
GW788388 treatment regulates the expression of cardiogenic markers in the heart of mice chronically infected with *T*. *cruzi*. Mice were infected with *T*. *cruzi* from the Colombian strain (10^2^ parasites). Treatment with GW788388 started on 120 dpi until 150 dpi in two administration schemes: once a week (GW1x) and three times a week (GW3x) followed up until 180 dpi. Non-infected (NI) mice were monitored as a control group. GATA-4, GATA-6, Nkx2-5, Tbox-5, troponin T, titin and desmin (A-G) mRNA levels relative to GAPDH levels from hearts of infected mice (2^−ʎʎCt^ values, fold change) are plotted. Sca-1^+^ cells (stained in red, arrows), a marker for cardiac regeneration, are detected in the heart of non-infected mice and mice at 120, 150 and 180 dpi, treated or not with GW one (GW1x) or three times a week (GW3x) by immunofluorescence assay (H). Significant differences between infected and non-infected groups are indicated by ^$ $ $^P<0.001, ^$ $^P<0.01 and ^$^P<0.05 and differences between infected mice GW788388 treated or not are indicated by *P<0.05. n = 5–6 mice per group.

Next, we checked whether the effect of this therapy on reversion of heart fibrosis was associated with induction of heart recovery through recruitment or differentiation of cardiomyocytes. Thus, we investigated the mRNA levels of cardiac cell markers such as GATA-4, GATA-6, Nkx2-5, Tbox-5, troponin T, titin and desmin (Fig 7A–7G). Almost all cardiac markers presented significantly reduced expression after *T*. *cruzi* infection, except for Nkx2-5 and desmin (Fig 7A–7G). At 150 dpi, only GW3x -treatment significantly increased GATA-6 and Tbox-5 expression in the cardiac tissue of chronically *T*. *cruzi*-infected mice (Fig 7B and 7D).

In order to confirm the cardiac recovery process, we also analyzed the stem cell antigen-1 (Sca-1^+^), a marker for cardiac stem cell, directly in the heart tissue [37]. The presence of cells Sca-1^+^ was rare in the heart of non-infected mice and after 120 dpi. At 150 dpi, Sca-1^+^ cells were observed in the heart tissue, being more evident in infected mice treated with GW3x (Fig 7H, white arrows), supporting that GW788388 therapy stimulated the arrival of stem cells with cardiac phenotype. At 180 dpi, 30 days after treatment interruption, the presence of Sca-1^+^ cells were still observed in the group of mice treated with GW3x from 120 to 150 dpi (Fig 7H, white arrows). Altogether, the increased levels of GATA-6 and Tbox-5 mRNA and the presence of Sca-1^+^ cells suggested the emergence of cells with high potential of cardiac phenotype which could indicate cardiac recovery.

### GW788388 administration interfere on the inflammatory profile

**Fig 8:**
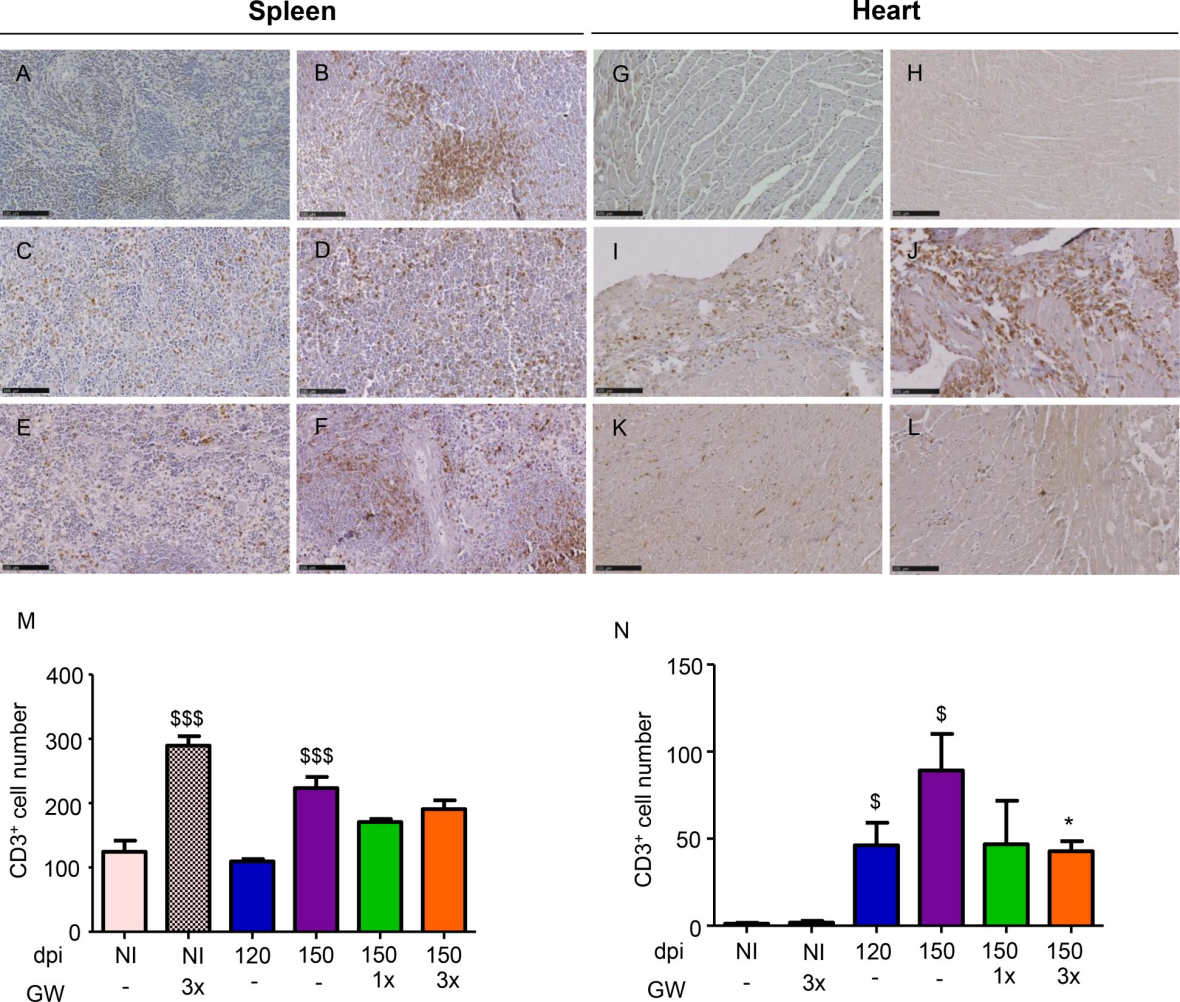
GW788388 treatment decreases CD3^+^ cells number in the heart of mice chronically infected with *T*. *cruzi*. Mice were infected with *T*. *cruzi* from the Colombian strain (10^2^ parasites). Treatment with GW788388 orally started on 120 dpi until 150 dpi in two administration schemes: once a week (GW1x) and three times a week (GW3x) followed up until 150 dpi. Non-infected (NI) mice were monitored as a control group. Spleen (A-F) and heart (G-L) sections were stained for CD3^+^: (A, G) Non-infected non-treated mice, (B, H) Non-infected treated mice, non-treated infected mice at (C, I) 120 dpi, and (D, J) 150 dpi; infected mice at 150 dpi treated with GW788388 (E, K) GW1x, and (F, L) GW3x. Graphs of the number of CD3^+^ cells in the (M) spleen and (N) heart are shown. Data represent mean± standard deviation of at least three mice per group in three independent experiments. Significant differences between infected and non-infected groups are indicated by ^$ $ $^P<0.001, and ^$^P<0.05 and differences between infected mice GW788388 treated or not are indicated by *P<0.05.

During the chronic phase, inflammatory infiltrates and *T*. *cruzi* antigens are observed in the heart tissue, both processes could lead to cardiac damage and prominent fibrosis. The inflammatory infiltrate is composed mainly of T cells [29,32,34]. Thus, we also analyzed if the inhibition of TGF-β pathway could interfere on the inflammatory profile in our experimental model of chronic cardiomyopathy induced by *T*. *cruzi* infection. To this end, we assessed the presence of CD3^+^ cells in the heart and spleen tissues. Non-infected and GW-treated mice showed an increase in the number of CD3^+^ cells in the spleen, clearly demonstrating the potential of GW therapy on interfering on immune cells. Although this clear effect in avoiding the TGF-β control of T cells proliferation, GW treatment in the absence of *T*. *cruzi* infection did not altered the subset of memory T cells but decreased its activation profile, inhibiting the migration to the heart tissue as expected as no injury in the heart was observed. As already described [30,31] after *T*. *cruzi* infection, it was observed an increase in the frequency of CD3^+^ cells either in the spleen and in the heart (Fig 8). Interestingly, GW treatment three times a week decreases the frequency of CD3^+^ cells in the heart, which could indicate an effect in the migratory capacity of these cells from the spleen.

*T*. *cruzi* infection promotes splenomegaly in this mice model [30], in both acute and chronic phases, reproducing aspects of Chagas disease [31]. Here, we confirmed symptoms of spleen enlargement during the chronic infection and showed that GW treatment partially reversed this process (Table 2). Moreover, we investigated the CD4^+^ and CD8^+^ cell profile in the spleen of studied mice: naive, T central memory (TCM) and T effector memory (TEM). We observed that *T*. *cruzi* chronic infection increased CD8^+^ cells from all CD8^+^ subtypes: CD44^high+^/CD62L^-^ (TEM), CD44^high-^/CD62L^+^ (naïve) and CD44^high+^/CD62L^+^ (TCM). Regarding the CD4^+^ cells, no difference was observed, but analysis of the different CD4^+^ subtypes showed that CD44^high+^/CD62L^-^, CD44^high-^/CD62L^+^ and CD44^high+^/CD62L^+^ were increased (Table 2). The GW788388 treatment did not alter the increased expression of CD8^+^ cell profiles, but GW1x treatment showed a tended to decrease the number of these cells (p = 0.053). We also observed that GW1x treatment decreased the frequency of CD4^+^/CD44^high-^/CD62L^+^, CD4^+^/CD44^high+^/CD62L^+^, CD8^+^/ CD44^high-^/CD62L^+^, CD8^+^/ CD44^high+^/CD62L^-^ and CD8^+^/ CD44^high+^/CD62L^+^, and GW3x only modulated the TCM CD4^+^/CD44^high+^/CD62L^+^ (Table 2). Among the activation molecules evaluated (CD49d, CD11a and CD45R), both T lymphocytes, CD4+ and CD8+, have increased activation profile with *T*. *cruzi* infection. Moreover, both schemes of GW treatments decreased this activation profile in at least one of the studied molecules (Table 2).

**Table 2:** Inflammatory profile.

	NI	NI+GW	Tc 150	Tc+GW1x	Tc+GW3x
**Relative spleen weight** (mg/g)	4.2±0.9	4.1±1.5	20.6±3.7^$ $ $^	13.1±6.4*	14.1±2.8*
**CD4**^**+**^ **subsets (number)**	29.4±6.1	29.5±9.7	26.8±9.6	29.4±7.9	23.8±7.8
CD44^high-^/CD62L^+^	4.6±0.2	4.8±0.9	8.6±0.2^$^	4.3±1.2*	8.7±3.5
CD44^high+^/CD62L^-^	20.6±0.2	18.9±1.7	26.8±0.1^$^	25.7±7.1	36.5±13.9
CD44^high+^/CD62L^+^	3.1±0.1	3.5±1.2	6.4±0.3^$ $^	3.8±0.3**	5.7±1.8*
CD49d	11.3±5.3	0.8±0.5*	7.2±2.5	2.8±1.6*	4.7±0.1
CD11a	4.6±1.9	5.4±4	15.1±5.4^$^	14.3±1.8	8.1±3.6*
CD45R	2.3±0.7	5.4±4.1	8.8±4.9^$ $^	5.1±2.1	2.4±0.5**
**CD8^+^ subsets (number)**	12.5±8.4	10.3±4.4	18.3±5.4^$ $^	14.1±6.5	17.2±9.4
CD44^high-^/CD62L^+^	1.5±0.1	3.6±0.5	10.3±0.3^$ $ $^	6.0±0.3**	9.4±3.8
CD44 ^high+^/CD62L^-^	13.8±0.1	16.5±4.7	24.5±0.8^$^	11.0±1.4*	29.6±14.2
CD44 ^high+^/CD62L^+^	2.9±0.1	8.2±4.3	18.1±0.1^$^	12.5±4.2*	14.2±5.4
CD49d	6.7±3.2	0.8±0.5*	8.5±3.5	3.7±1.6*	4.1±2.2
CD11a	12.7±5.9	2.5±2*	13.5±7.7	11.1±0.5	8.2±4.4
CD45R	4.2±1.2	6.5±1.9	6.1±1.3^$^	4.7±2.5	3.4±1.2**

Relative spleen weight and CD4^+^ and CD8^+^ subset profiles in non-infected mice (NI) and in Chronically *T*. *cruzi* (Tc)-infected mice. Significant differences between infected and non-infected groups are indicated by ^$ $ $^P< 0.001, ^$ $^P< 0.01, ^$^P< 0.05 and differences between infected mice GW788388 treated or not are indicated by **P<0.01 and *P< 0.05. n = 8–10 mice per group.

## Discussion

TGF-β is notably one of the main cytokines involved in the process of fibrosis [38,39]. Our previous work demonstrated the protective effect of oral administration of GW788388, an inhibitor of TGF-β receptor activity, at 20 dpi in an acute model of experimental CD by decreasing cardiac fibrosis and improving overall survival [19]. In the present work, we aimed to study the potential use of GW788388 in a pre-clinical chronic Chagas cardiac fibrosis model, which corresponds to the most frequent situation observed in humans. To test this hypothesis, we used a well-established pre-clinical mouse model of chronic *T*. *cruzi* infection, which reproduces relevant clinical features of chagasic heart disease [29–32,40]. Our data demonstrated that treatment with GW788388 initiated at 120 dpi, when CCC is already established, improved heart rate, decreased AVB II events, triggered correction of Cx43 intercellular plaques, reversed cardiac fibrosis, electrical abnormalities and restored a normal LVEF. Interestingly, GW788388 treatment was able to interfere in the MMP/TIMP pathway and regenerate heart tissue with input of new cardiac cells.

Chronic chagasic cardiomyopathy is accompanied by focal fibrosis, which can be responsible for malignant arrhythmias with electrical conduction disturbances and heart failure. This clinical feature is associated with ~4% annual mortality [2]. An excess of scar formation due to elevated production and deposition of extracellular matrix, compromising organ functioning, is typical for the cardiac fibrotic process observed in CCC, with possible fatal outcome [2]. The therapeutic approaches for the management of CCC still follow the standard recommendations for treatment of heart failure due to other clinical conditions. There is no effective treatment available for the chronic phase and patients with CCC have a shortened life expectancy [41]. Treatments do not focus specifically on heart failure originating from CD but there are some peculiarities of CCC disease, which deserves specific therapeutic approaches.

Beneficial effects of Bz on heart fibrosis due to CD has been demonstrated [34,42–44]. Thus, recently, an attempt to understand Bz efficacy during the chronic phase of CD was performed in a multicenter clinical trial (BENEFIT). However, despite the observation of decreased parasite load, few prominent clinical benefits were obtained, at least during the first 5 years of follow up [20].

Excessive TGF-β may be deleterious by promoting extracellular matrix deposition, increased myocardial stiffness and diastolic dysfunction [3]. Here we showed a two- fold increase in circulating TGF-β levels, which is consistent with an increase in phosphorylated Smad-2/3 in the heart tissue, corroborating data found in the cardiac tissue of CD patients with cardiomyopathy [8]. TGF-β is a pleiotropic cytokine with a diversity of regulatory functions. Thus, a baseline level of TGF-β signaling is possibly necessary to preserve cardiac structure and to protect the pressure-overloaded myocardium from uncontrolled matrix degradation, which may result in cardiac dilatation. This is an important observation that prompted us to test three therapeutic schemes: single dose, one dose per week, and three doses per week during four weeks, seeking to reduce the possibility of negative side effects in case of complete TGF-β blockade. ECG methods help to predict, in a non-invasive manner, a worse prognosis in the animals, which develop malignant ventricular arrhythmias. We observed that the single dose therapy was only partially effective, just reducing the interval of P duration, with no effect on the other ECG parameters. Thus, we focused on treating *T*. *cruzi*-infected mice using GW788388 with either one or three doses per week, for four weeks from 120 dpi onwards, with final analysis of the animals at 150 dpi.

As a pleiotropic protein, TGF-β was already described to regulate connexin-43 plaque formation. In 2009, Chi Qiang et al reported that cells treated with TGF-β reduced the connexin-43 expression [45]. Here, we observed that the higher levels of TGF-β activity in an experimental model of Chagas disease chronic phase, with C57BL/6 mice infected with Colombian strain of *T*. *cruzi*, was followed by a punctate, diffuse and non-uniform cardiac pattern of Cx43 staining, corroborating previous data in mouse models [19,29,46] and non-human primates [47] and patients with cardiac damage due to chronic Chagas disease [8]. Importantly, after the different therapeutic schemes using GW788388, mice showed a preserved staining for Cx43 plaques in the heart that may contribute to a better cardiac electrical conduction. In the heart, gap junctions such as Cx43 plaques may mediate electrical current flow, thereby coordinating the spread of excitation and subsequent contraction throughout the myocardium [35].

ECG data showed that, at 150 dpi, non-treated infected mice had a worse cardiac electrical and functional alterations when compared to the same group pre-therapy (at 120 dpi), as observed by an increase in heart rate frequency, reduction of P duration and prolonged PR and QTc intervals. Therefore, our data indicate that inhibition of the TGF-β pathway avoided the increase of heart abnormalities. Moreover, GW788388 therapy restored cardiac function, increasing LV volume and improving LVEF. We showed that late TGF-β inhibition starting at 120 dpi exerts beneficial actions through attenuation of cardiac fibrosis as observed by collagen type I and fibronectin lower expression. However, we could not exclude potential effects of lower TGF-β interfering with other inflammatory cytokines. Recently, Vilar-Pereira et al. [34] reported that the combination of an immunoregulator compound, pentoxifylline, with a suboptimal dose (25 mg/Kg/day) of the trypanocidal drug Bz reduced heart fibrosis with lower cardiac parasite load and parasitemia, minimizing heart electrical dysfunction. This combinatorial therapy was important for repositioning of the immune response reducing the up-regulated TNF/TNFR1 and iNOS/NO axis and controlling parasite replication.

The positive scenario obtained after GW788388 treatment with reduced levels of circulating TGF-β, lower phosphorylation of Smad 2/3 and reversion of deposition of ECM proteins in the heart, as well as improved heart rate and lower QTc interval, may contribute to a better function of the heart reflecting in a normal left ventricular ejection fraction. In our chronic model of CD, it seems that the balance between MMPs and their tissue inhibitors, TIMPs, is pivotal in the remodeling of ECM deposition and reduction of fibrosis.

GW788388 is possibly acting through regulation of metaloproteinases -2 and -9. MMP-2 and -9 are increased in the serum of patients with the cardiac form of CD [48], but, in the present study, we investigated the MMPs transcription in the heart tissue of chronically *T*. *cruzi*- infected mice and observed opposite results. Collagen turnover depends on a balance between its production and degradation mainly mediated by MMPs and inhibition by TIMP. A potential mechanism of MMP expression control due to GW788388 action, is the significant decrease in expressions of TIMP-1, -2 and -4 protein. It is known that TGF-β regulates both MMPs expression and activity by inducing higher expression of TIMPs [49,50]. TGF-β blockage by GW788388 in our model clearly induces a decrease in TIMPs and favoring MMPs expression, with probable activity on degrading fibrotic areas in the heart, as a significant reduction of ECM proteins expression was clearly detected.

Transcription factors govern most developmental gene expression programs and can completely alter the state of a differentiated cell [51,52]. In the heart, a large group of conserved transcription factors cooperates to control cardiac gene expression, including members of the Nkx, GATA, Islet, Tbx, Mef2 and Hand families [1]. These transcription factors could control cardiac cell genes involved in contraction and morphology, contributing to cardiogenesis [5]. In the present study, we demonstrated that mice chronically infected with *T*. *cruzi* treated with GW788388 thrice a week for 30 days presented increased expression of cardiogenic markers such as GATA-6 and Tbox-5, implicating that TGF-β inhibitors present a therapeutic potential in regenerative cardiovascular medicine.

Recently, a study using a model of heart injury of myocardial infarction showed that TGF-β receptor inhibition enhanced cardiac recovery and improved cardiac function with involvement of Nkx2.5 [52]. Further, specific combination of three transcription factors (GATA, Mef2c, and Tbox-5) was sufficient to generate functional beating cardiomyocytes, which were globally reprogrammed to adopt a cardiomyocyte-like gene expression profile [53,54].

To verify the evolution of the observed beneficial effects of the TGF-β inhibition, we followed up two different groups of *T*. *cruzi* infected mice: non-treated and GW3x treated until 180 dpi, after treatment interruption at 150 dpi. GW3x-treated group tend to remain in normal condition even after 30 days of treatment interruption. The data was not statistically analyzed due to the small sample size. Interestingly, regarding the cardiac markers, the GW3x-treated group at 180 dpi tend to present increased gene expression not only for GATA-6 and T-Box5, but also GATA-4, Troponin T, Titin and Desmin makers. These data suggested that the treatment with GW3x, could stimulate the cardiac recovery even after treatment interruption. Finally, it would be interesting to verify the heart zone with cardiac recovery, the origin of cells differentiated into cardiomyocytes and the localization of studied molecules: active MMPs and TIMPs.

After injury, cardiac progenitor cells can be activated and may differentiate into new myocytes. Cardiac progenitor cells show mixed and overlapping expression of stem cell markers, such as stem cell antigen-1 [54]. A study isolated Sca-1 cells from adult mice hearts and after oxytocin treatment the Sca-1^+^ cells started to express genes of cardiac transcription factors and showed sarcomeric structure and spontaneous beating, suggesting that Sca-1^+^ cells may contribute to the regeneration of injured hearts [55]. Here, we elucidated that the GW788388 treatment during the chronic phase of an experimental model of Chagas’ heart disease stimulated the expression of Sca-1 in the heart tissue, suggesting that after the damage and treatment we observed cardiac recovery.

*T*. *cruzi* infection triggers an immune response that plays an important role in the control of parasite growth during the acute and chronic phases and the development of the disease [56]. As an intracellular pathogen, *T*. *cruzi* triggers a CD4^+^ and CD8^+^ T-cell response [31,57,58].T cells were already described as an important actor during the development of chronic chagasic myocarditis. The inflammatory infiltrate is composed mostly of T cells, predominantly CD8^+^ (cytotoxic/ suppressor) [30, 59]. Here, we analyzed if the TGF-β pathway blockage interferes with CD3^+^ cells in inflammatory infiltrates in the heart and spleen tissues. We observed that GW788388 administration three times a week decreased the influx of CD3^+^ cells to the heart tissue. Besides that, the chronic infection increased the frequency of naive, central, memory and effector memory T cells, up-regulated the frequency of cell adhesion molecule-bearing cells and increased cytokine (TGF-β) production. Altogether, this process leads to increase of lymphocytes adhesion to endothelium and migration into the extravascular space [60] of inflamed tissues. It could explain the increase of numbers of CD3^+^ lymphocytes in the heart during the chronic disease. Meanwhile, the treatment with GW788388 decreased the expression, at least, of one of the cell adhesion molecules analyzed (CD49d, CD11a and CD45R), supporting the lowest numbers of CD3^+^ lymphocytes in the heart. Thus, the regulation and repositioning of the immune response during the chronic phase of *T*. *cruzi* infection could be an alternative mechanism for the interruption of disease progression, with possibility to reverse the CCC, favoring cardiac electrical and functional performance.

### Concluding remarks

This important pre-clinical proof of concept study places TGF-β regulator compounds as an alternative strategy for the treatment of heart fibrosis, once GW788388 treatment improved ECG and ECO profiles; modulated TGF-β levels and its intracellular proteins; reversed the loss of connexin-43 intercellular plaques; reduced fibrosis of the cardiac tissue; restored cardiac recovery markers and reduced the migration of CD3^+^ cells to the heart. Furthermore, at 180 dpi, 30 days after treatment interruption, the GW3x-treated group remained in a better cardiac functional condition. These data are promising and the inhibition of TGF-β pathway could be an important and relevant strategy of therapy for cardiac fibrosis during the chronic phase of Chagas’ heart disease.

## Supporting information

S1 Fig ECG analysis demonstrating abnormal cardiac electrical impulse in mice chronically infected with *T*. *cruzi*. Mice were infected with *T*. *cruzi* from the Colombian strain (102) and were accompanied for 120 days post infection. Non-infected (NI) mice were monitored as a control group. Barr graphs represents mean± SD of ECG parameters: bpm (A); PR interval in milliseconds (B); P wave duration in milliseconds (C); QRS interval in milliseconds (D); QT interval in milliseconds (E); corrected QT interval in milliseconds (F); Representative ECG tracings of non-infected mice and infected mice at 120 dpi. Note the incidence of arrhythmia; atrioventricular block (AVB1 and AVB2) and fibrillation disorders (G) and % of arrhythmia afflicted mice (H). Asterisk indicates significant difference between infected and non-infected groups (***P< 0.001). n = ~18 mice per group.
(TIF)

S2 Fig ECG analysis demonstrating abnormal cardiac electrical impulse in mice chronically infected with *T*. *cruzi*. Mice were infected with *T*. *cruzi* from the Colombian strain (10^2^) and were observed at 150 and 180 days post infection. Treatment with GW788388 orally started on 120 dpi until 150 dpi three times a week (GW3x) followed up until 150 dpi. Barr graphs represents mean± SD of ECG parameters: bpm (A); PR interval in milliseconds (B); P wave duration in milliseconds (C); QRS interval in milliseconds (D); corrected QT interval in milliseconds (E). n = ~18 mice per group, except for mice from 180 dpi which n = 2–3.
(TIF)

## References

[1] Alexander J, Bruneau B. Lessons for cardiac regeneration and repair through development. Trends Mol Med. 2010;16: 426–434. 10.1016/j.molmed.2010.06.003 20692205 PMC3089764

[2] Rassi A, Rassi A, Marin-Neto JA. Chagas heart disease: Pathophysiologic mechanisms, prognostic factors and risk stratification. Mem Inst Oswaldo Cruz. 2009;104: 152–158. 10.1590/s0074-02762009000900021 19753470

[3] Dobaczewski M, Chen W, Frangogiannis NG. Transforming growth factor (TGF)-β signaling in cardiac remodeling. J Mol Cell Cardiol. Elsevier Ltd; 2011;51: 600–606. 10.1016/j.yjmcc.2010.10.033 21059352 PMC3072437

[4] Gramley F, Lorenzen J, Koellensperger E, Kettering K, Weiss C, Munzel T. Atrial fibrosis and atrial fibrillation: The role of the TGF- β 1 signaling pathway. Int J Cardiol. Elsevier Ireland Ltd; 2010;143: 405–413. 10.1016/j.ijcard.2009.03.110 19394095

[5] Olson EN. Gene regulatory networks in the evolution and development of the heart. Science (80-). 2006;29: 1922–1927. 10.1126/science.1132292 17008524 PMC4459601

[6] Robertson IB, Rifkin DB. Unchaining the beast; insights from structural and evolutionary studies on TGFβ secretion, sequestration, and activation. Cytokine Growth Factor Rev. Elsevier Ltd; 2013;24: 355–372. 10.1016/j.cytogfr.2013.06.003 23849989 PMC3780968

[7] Massague J. A very private TGF-beta receptor embrace. Mol Cell. 2008;29: 149–150. 10.1016/j.molcel.2008.01.006 18243107

[8] Araujo-Jorge TC, Waghabi MC, Hasslocher-moreno AM, Xavier S, Higuchi MDL, Keramidas M, et al Implication of Transforming Growth Factor–b 1 in Chagas Disease Myocardiopathy. J Infect Dis. 2002;186: 1823–1828. 10.1086/345882 12447769

[9] Araújo-jorge TC, Waghabi MC, Bailly S, Feige J. The TGF-β pathway as an emerging target for Chagas disease therapy. Clin Pharmacol Ther. 2012;92: 613–621. 10.1038/clpt.2012.102 22990752

[10] Silva J, Twardzik D, Reed S. Regulation of Trypanosoma cruzi infections in vitro and in vivo by transforming growth factor beta (TGF-beta). J Exp Med. 1991;174. 10.1084/jem.174.3.539 PMC2118925 1908509

[11] Pérez AR, Silva-barbosa SD, Berbert LR, Revelli S, Beloscar J, Savino W, et al Immunoneuroendocrine alterations in patients with progressive forms of chronic Chagas disease. J Neuroimmunol. Elsevier B.V.; 2011;235: 84–90. 10.1016/j.jneuroim.2011.03.010 21496931

[12] Ferreira RR, Souza EM de, Oliveira FL de, Ferrão PM, Gomes LHF, Lima LM-, et al Proteins involved on TGF-β pathway are up- regulated during the acute phase of experimental Chagas disease. Immunobiology. Elsevier GmbH.; 2016;221: 587–94. 10.1016/j.imbio.2016.01.009 26852285

[13] Calzada JE, Beraún Y, González CI, Martín J. Cytokine Transforming growth factor beta 1 (TGF b 1) gene polymorphisms and Chagas disease susceptibility in Peruvian and Colombian patients. Cytokine. Elsevier Ltd; 2009;45: 149–153. 10.1016/j.cyto.2008.11.013 19136278

[14] Ferreira RR, Madeira S, Alves GF, Chambela C, Oliveira E De, Curvo V, et al TGF-β Polymorphisms Are a Risk Factor for Chagas Disease. Dis Markers. 2018;2018: 1–10. 10.1155/2018/4579198 PMC5835243 29670670

[15] Saraiva RM, Waghabi MC, Vilela MF, Madeira FS, Silva GMS da, Xavier SS, et al Predictive value of transforming growth factor-β1 in Chagas disease: towards a biomarker surrogate of clinical outcome. Trans R Soc Trop Med Hyg. 2013;107: 518–525. 10.1093/trstmh/trt050 23787193

[16] Waghabi MC, Keramidas M, Bailly S, Degrave W, Mendonça-Lima L, Soeiro M de NC, et al Uptake of Host Cell Transforming Growth Factor- by Trypanosoma cruzi Amastigotes in Cardiomyocytes. Am J Pathol. 2005;167: 993–1003. 10.1016/s0002-9440(10)61189-3 16192635 PMC1603686

[17] Waghabi MC, Keramidas M, Calvet CM, Meuser M, Nazare M De, Soeiro C, et al SB-431542, a transforming growth factor beta inhibitor, impairs Trypanosoma cruzi infection in cardiomyocytes and parasite cycle completion. Antimicrob Agents Chemother. 2007;51: 2905–2910. 10.1128/AAC.00022-07 17526757 PMC1932517

[18] Waghabi MC, Souza EM De, Oliveira GM De, Keramidas M, Feige J, Araujo-Jorge TC, et al Pharmacological inhibition of transforming growth factor beta signaling decreases infection and prevents heart damage in acute Chagas’ disease. Antimicrob Agents Chemother. 2009;53: 4694–4701. 10.1128/AAC.00580-09 19738024 PMC2772341

[19] de Oliveira FL, Souza EM De, Oliveira GM De, Arau TC, Degrave WM, Feige J, et al Oral Administration of GW788388, an Inhibitor of Transforming Growth Factor Beta Signaling, Prevents Heart Fibrosis in Chagas Disease. PLoS Negl Trop Dis. 2012;6: e1696 10.1371/journal.pntd.0001696 22720109 PMC3373641

[20] Morillo CA, Marin‑Neto J.A., Avezum A., Sosa‑Estani S, Rassi A Jr, Rosas F, et al Randomized Trial of Benznidazole for Chronic Chagas’ Cardiomyopathy. N Engl J Med. 2015; 373 (14):1295–1306. 10.1056/NEJMoa1507574 26323937

[21] Rossi MA. Fibrosis and inflammatory cells in human chronic chagasic myocarditis: scanning electron microscopy and immunohistochemical observations. Int J Cardiol. 1998;66: 183–194. 9829333 10.1016/s0167-5273(98)00208-3

[22] Cardillo F, de Pinho RT, Antas PRZ, Mengel J. Immunity and immune modulation in Trypanosoma cruzi infection. Pathog Dis. 2015;73: ftv082 10.1093/femspd/ftv082 26438729 PMC4626602

[23] Rassi A Jr, Rassi SG, Rassi A. Sudden Death in Chagas ‘ Disease. Arq Bras Cardiol. 2001;76: 75–96. 10.1590/s0066-782x2001000100008 11175486

[24] Robertl RR, Martinezf EE, Andradef JL, Araujof VL, Britof FS, Portugalf OP. Chagas cardiomyopathy and captopril. Eur Hear J. 1992;13: 966–970. 10.1093/oxfordjournals.eurheartj.a060301 1644089

[25] Dávila D, Angel F, Arata de Bellabarba G, Donis J. Effects of metoprolol in chagasic patients with severe congestive heart failure. Int J Cardiol. 2002;85: 255–260. 12208592 10.1016/s0167-5273(02)00181-x

[26] Botoni FA, Poole-wilson PA, Sci FM, Ribeiro ALP. A randomized trial of carvedilol after renin-angiotensin system inhibition in chronic Chagas cardiomyopathy. Am Hear J. 2007;153: 1–8. 10.1016/j.ahj.2006.12.017 17383291

[27] Issa VS, Amaral AF, Cruz D, Ferreira SMA, Chizzola PR, Souza GEC, et al Blocker Therapy and Mortality of Patients With A Subanalysis of the REMADHE Prospective Trial. Circ Hear Fail. 2015;3: 82–88. 10.1161/CIRCHEARTFAILURE.109.882035 19933408

[28] Szajnbok FE, Barretto AC, Mady C, Parga Filho J, Gruppi C, Alfieri RG, et al Beneficial effects of enalapril on the diastolic ventricular function in Chagas myocardiopathy. Arq Bras Cardiol. 1993;60: 273–278. 8311739

[29] Pereira IR, Vilar-pereira G, Silva AA, Moreira OC, Britto C, Diana E, et al Tumor Necrosis Factor Is a Therapeutic Target for Immunological Unbalance and Cardiac Abnormalities in Chronic Experimental Chagas ‘ Heart Disease. Mediators Inflamm. 2014;2014: 1–16. 10.1155/2014/798078 PMC4130030 25140115

[30] Silverio J, Pereira I, Cipitelli MC, Vinagre N, Rodrigues M, Gazzinelli R, et al CD8 + T-Cells Expressing Interferon Gamma or Perforin Play Antagonistic Roles in Heart Injury in Experimental Trypanosoma Cruzi-Elicited Cardiomyopathy. PLoS Pathog. 2012;8: e1002645 10.1371/journal.ppat.1002645 22532799 PMC3330123

[31] Pereira IR, Vilar-Pereira G, Marques V, da Silva AA, Caetano B, Moreira OC, et al (2015) A Human Type 5 Adenovirus-Based Trypanosoma cruzi Therapeutic Vaccine Re-programs Immune Response and Reverses Chronic Cardiomyopathy. PLoS Pathog 11(1): e1004594 10.1371/journal.ppat.1004594 25617628 PMC4305326

[32] Pereira IR, Vilar-Pereira G, Moreira OC, Ramos IP, Gibaldi D, Britto C, et al Pentoxifylline Reverses Chronic Experimental Chagasic Cardiomyopathy in Association with Repositioning of Abnormal CD8 + T-Cell Response. PLoS Negl Trop Dis. 2015;9: 1–23. 10.1371/journal.pntd.0003659 25789471 PMC4366205

[33] Gellibert F, de Gouville A, Woolven J, Mathews N, Nguyen V, Bertho-Ruault C, et al Discovery of 4-{4-[3-(pyridin-2-yl)-1H-pyrazol-4-yl]pyridin-2-yl}-N-(tetrahydro-2H- pyran-4-yl)benzamide (GW788388): a potent, selective, and orally active transforming growth factor-beta type I receptor inhibitor. J Med Chem. 2006;49: 2210–2221. 10.1021/jm0509905 16570917

[34] Vilar-Pereira G, Resende P, de Souza RL, Cruz M, da Silva A, Britto C, Lannes-Vieira J. Combination of Chemotherapy with Suboptimal dose of Benznidazole and 5 Pentoxifylline Sustained the Partial Reversion of Experimental Chagas’ Heart Disease. Antimicrob Agents Chemother. 2016;60: 4297–309. 10.1128/AAC.02123-15 27161638 PMC4914640

[35] Spray DC, Burt JM. Structure-activity relations of the cardiac gap junction channel. Am J Physiol. 1990; 258 (2 Pt 1):C195–205. 1689543 10.1152/ajpcell.1990.258.2.C195

[36] Kisseleva T, Brenner DA. Mechanisms of Fibrogenesis. Exp Biol Med. 2008;233: 109–122. 10.3181/0707-MR-190 18222966

[37] Valente M, Nascimento DS, Cumano A, Pinto-do-Ó P. Sca-1 ^+^ Cardiac Progenitor Cells and Heart-Making: A Critical Synopsis. Stem Cells Dev. 2014;23: 2263–2273. 10.1089/scd.2014.0197 24926741 PMC4172562

[38] Pohlers D, Brenmoehl J, Löf I, Müller CK, Leipner C, Schultze-mosgau S, et al TGF- β and fibrosis in different organs—molecular pathway imprints. Biochim Biophys Acta. 2009;1792: 746–756. 10.1016/j.bbadis.2009.06.004 19539753

[39] Biernacka A, Dobaczewski M, Frangogiannis N. TGF- b signaling in fibrosis. Growth Factors. 2011;29: 196–202. 10.3109/08977194.2011.595714 21740331 PMC4408550

[40] Silverio J, De Oliveira Pinto L, da Silva A, de Oliveira G, Lannes-Vieira J. Perforin-expressing cytotoxic cells contribute to chronic cardiomyopathy in Trypanosoma cruzi infection. Int J Exp Pathol. 2010;91: 72–86. 10.1111/j.1365-2613.2009.00670.x 19878357 PMC2812730

[41] Nunes M, Dones W, Morillo C, Encina J, Ribeiro A. Council on Chagas Disease of the Interamerican Society of Cardiology. J Am Coll Cardiol. 2013;62: 767–76. 10.1016/j.jacc.2013.05.046 23770163

[42] Andrade S, Stocker-Guerret S, Pimentel A, Grimaud J. Reversibility of cardiac fibrosis in mice chronically infected with trypanosoma cruzi, under specific chemotherapy. Mem Inst Oswaldo Cruz. 1991;86: 187–200. 10.1590/s0074-02761991000200008 1842413

[43] Bustamante JM, Rivarola W, Fern AR, Enders JE, Fretes RE, Paglini-oliva P. Treatment with benznidazole or thioridazine in the chronic phase of experimental Chagas disease improves cardiopathy. Int J Antimicrob Agents. 2007;29: 733–737. 10.1016/j.ijantimicag.2007.01.014 17395432

[44] Caldas I, da Matta Guedes P, dos Santos F, de Figueiredo Diniz L, Martins T, da Silva do Nascimento, AF Azevedo M, et al Myocardial scars correlate with eletrocardiographic changes in chronic Trypanosoma cruzi infection for dogs treated with Benznidazole. Trop Med Int Heal. 2013;18: 75–84. 10.1111/tmi.12002 23107306

[45] Qiang C, Fenghai Z, Yangmin W. TGF-b1 inhibits connexin-43 expression in cultured smooth muscle cells of human bladder. Journal of Medical Colleges of PLA. 2009; 24: 283–287.

[46] Medeiros GA, Silverio JC, Marino APMP, Roffe E, Vieira V, Kroll-Palhares K, et al Treatment of chronically Trypanosoma cruzi -infected mice with a CCR1 / CCR5 antagonist (Met-RANTES) results in amelioration of cardiac tissue damage. Microbes Infect. 2009;11: 264–273. 10.1016/j.micinf.2008.11.012 19100857

[47] Carvalho CME, Silverio JC, Silva AA da, Pereira IR, Coelho JMC, Britto CC, et al Inducible Nitric Oxide Synthase in Heart Tissue and Nitric Oxide in Serum of Trypanosoma cruzi-Infected Rhesus Monkeys: Association with Heart Injury. PLoS Negl Trop Dis. 2012;6: e1644 10.1371/journal.pntd.0001644 22590660 PMC3348164

[48] Medeiros NI, Fares RCG, Franco EP, Sousa GR, Mattos RT, Chaves AT, et al Differential Expression of Matrix Metalloproteinases 2, 9 and Cytokines by Neutrophils and Monocytes in the Clinical Forms of Chagas Disease. PLoS Negl Trop Dis 2017; 11(1): e0005284 10.1371/journal.pntd.0005284 28118356 PMC5261563

[49] Bujak M, Frangogiannis NG. The role of TGF- β signaling in myocardial infarction and cardiac remodeling. Cardiovasc Res. 2007;74: 184–195. 10.1016/j.cardiores.2006.10.002 17109837 PMC1924687

[50] Doré LC, Crispino JD. Transcription factor networks in erythroid cell and megakaryocyte development. Blood. 2011;118: 231–239. 10.1182/blood-2011-04-285981 21622645 PMC3138678

[51] Lentjes M, Niessen H, Akiyama Y, de Bruïne A, Melotte V, van Engeland M. The emerging role of GATA transcription factors in development and disease. Expert Rev Mol Med. 2016;18: 1–15. 10.1017/erm.2016.2 26953528 PMC4836206

[52] Chen W, Liu Y, Ho Y, Wu SM. Pharmacological inhibition of TGFβ receptor improves Nkx2.5 cardiomyoblast-mediated regeneration. Cardiovasc Res. 2015;105: 44–54. 10.1093/cvr/cvu229 25362681 PMC4342671

[53] Ieda M, Fu J, Delgado-olguin P, Vedantham V, Hayashi Y, Bruneau BG, et al Direct Reprogramming of Fibroblasts into Functional Cardiomyocytes by Defined Factors. Cell. Elsevier Ltd; 2010;142: 375–386. 10.1016/j.cell.2010.07.002 20691899 PMC2919844

[54] Le T, Chong J. Cardiac progenitor cells for heart repair. Cell Death Discov. The Author(s); 2016;2: 16052 10.1038/cddiscovery.2016.52 27551540 PMC4979410

[55] Matsuura K, Nagai T, Nishigaki N, Oyama T, Nishi J, Wada H, et al Adult Cardiac Sca-1-positive Cells Differentiate into Beating Cardiomyocytes. J Biol Chem. 2004;279: 11384–11391. 10.1074/jbc.M310822200 14702342

[56] Meis J De, Morrot A, Savino W. Differential Regional Immune Response in Chagas Disease. 2009;3 10.1371/journal.pntd.0000417 PMC2700264 19582140

[57] Tarleton RL, Koller BH, Latour A, Postan M. Susceptibility of beta 2-microglobulin-deficient mice to Trypanosoma cruzi infection. Nature. 1992;355: 242–244. 10.1038/355242a0 1549177

[58] Tarleton RL, Grusby MJ, Postan M, Glimcher LH. Trypanosoma cruzi infection in MHC-deficient mice: further evidence for the role of both class I-and class ll-restricted T cells in immune resistance and disease. Int Immunol. 1996;8: 13–22. 10.1093/intimm/8.1.13 8671585

[59] Higuchi M, Gutierrez P, Aiello V, Palomino S, Bocchi E, Kalil J, et al Immunohistochemical characterization of infiltrating cells in human chronic chagasic myocarditis: Comparison with myocardial rejection process. Virchows Arch A Pathol Anat Histopathol. 1993;423: 157–160. 10.1007/BF01614765 7901937

[60] Dettmar K, Carsten IS, Petra L, Diane S, Judith S. Transient lymphocyte decrease due to adhesion and migration following catumaxomab (anti-EpCAM x anti-CD3) treatment in vivo. Clin Transl Oncol. 2012;14: 376–381. 10.1007/s12094-012-0811-5 22551544

