# Supplementary figures and images for: TGF-β inhibitor therapy decreases fibrosis and stimulates cardiac improvement in a pre-clinical study of chronic Chagas’ heart disease
Source: PLoS Negl Trop Dis. 2019 Jul 31;13(7):e0007602. doi: 10.1371/journal.pntd.0007602 (PMC6690554; doi:10.1371/journal.pntd.0007602)

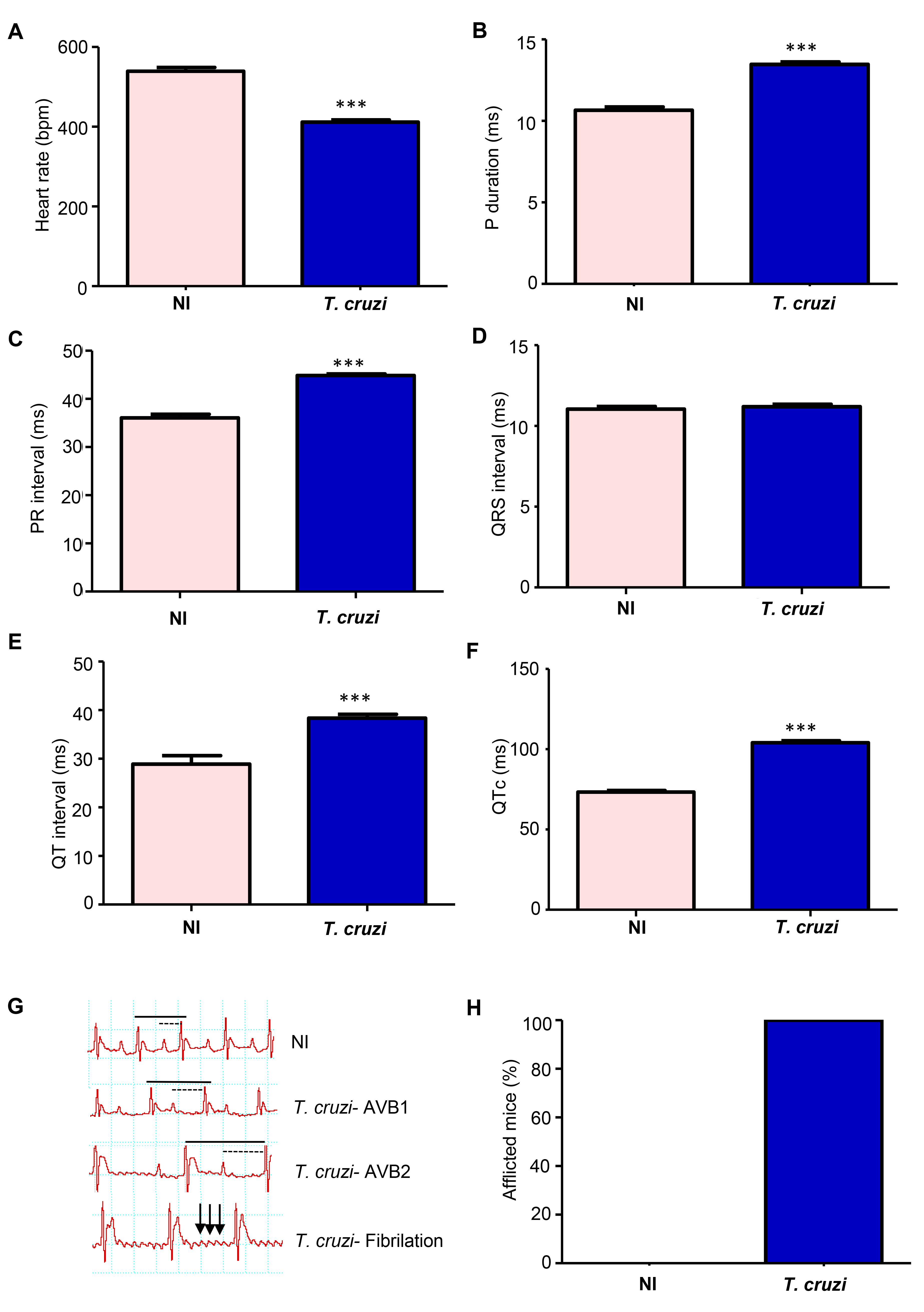

Supplement: S1 Fig — Mice were infected with T. cruzi from the Colombian strain (102) and were accompanied for 120 days post infection. Non-infected (NI) mice were monitored as a control group. Barr graphs represents mean± SD of ECG parameters: bpm (A); PR interval in milliseconds (B); P wave duration in milliseconds (C); QRS interval in milliseconds (D); QT interval in milliseconds (E); corrected QT interval in milliseconds (F); Representative ECG tracings of non-infected mice and infected mice at 120 dpi. Note the incidence of arrhythmia; atrioventricular block (AVB1 and AVB2) and fibrillation disorders (G) and % of arrhythmia afflicted mice (H). Asterisk indicates significant difference between infected and non-infected groups (***P< 0.001). n = ~18 mice per group. (TIF) [file pntd.0007602.s001.tif]

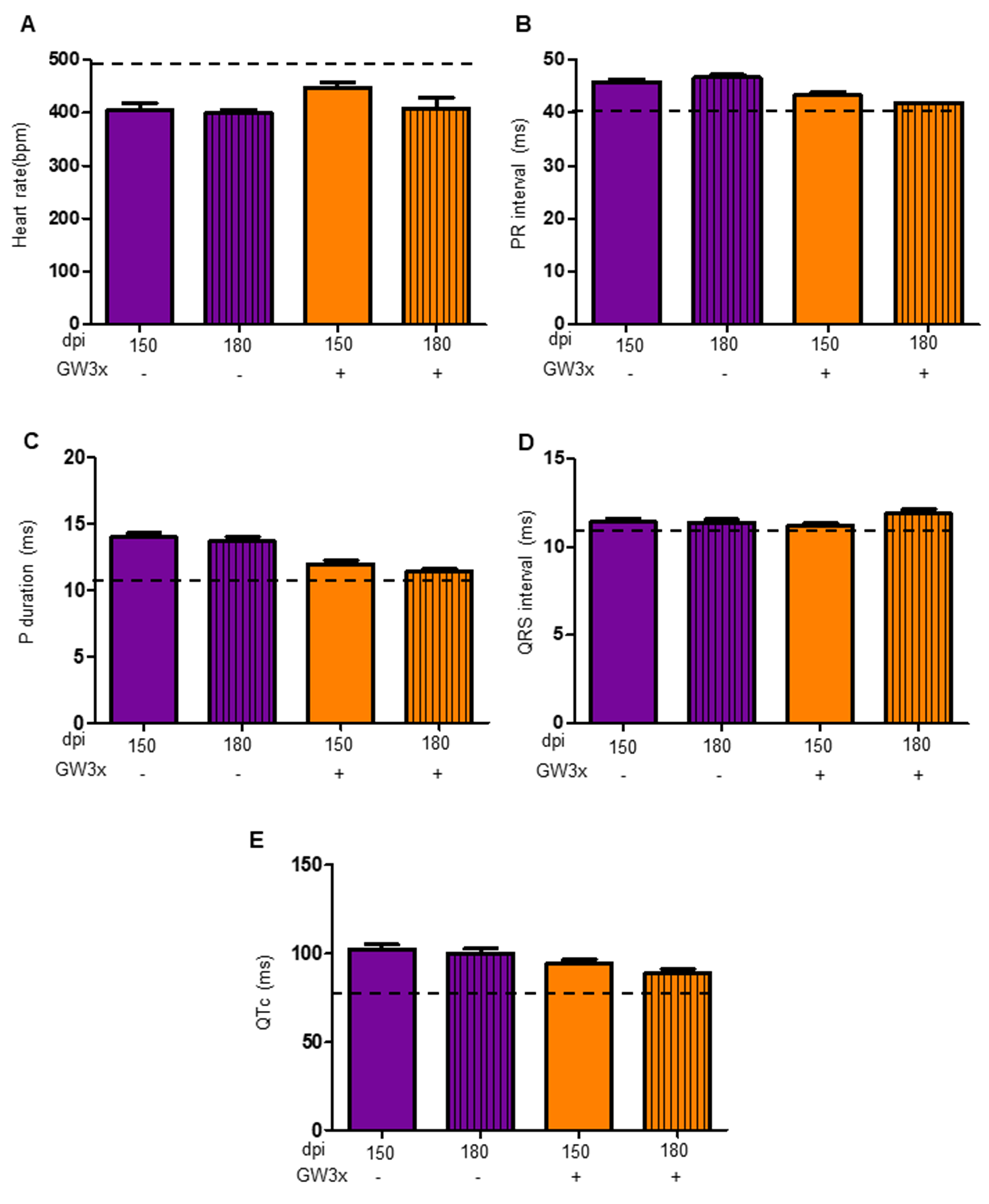

Supplement: S2 Fig — Mice were infected with T. cruzi from the Colombian strain (102) and were observed at 150 and 180 days post infection. Treatment with GW788388 orally started on 120 dpi until 150 dpi three times a week (GW3x) followed up until 150 dpi. Barr graphs represents mean± SD of ECG parameters: bpm (A); PR interval in milliseconds (B); P wave duration in milliseconds (C); QRS interval in milliseconds (D); corrected QT interval in milliseconds (E). n = ~18 mice per group, except for mice from 180 dpi which n = 2–3. (TIF) [file pntd.0007602.s002.tif]
